# Potential candidates from a functional food *Zanthoxyli Pericarpium* (Sichuan pepper) for the management of hyperuricemia: high-through virtual screening, network pharmacology and dynamics simulations

**DOI:** 10.3389/fendo.2024.1436360

**Published:** 2024-12-11

**Authors:** Meilin Chen, Xiaomei Chen, Qinghong Chen, Chenyang Chu, Shuxuan Yang, Chuanghai Wu, Yanting You, Andrew Hung, Angela Wei Hong Yang, Xiaomin Sun, Lin Zhou, Xiaoshan Zhao, Hong Li, Yanyan Liu

**Affiliations:** ^1^ School of Traditional Chinese Medicine, Southern Medical University, Guangzhou, China; ^2^ School of Science, STEM College, RMIT University, Melbourne, VIC, Australia; ^3^ School of Health and Biomedical Sciences, STEM College, RMIT University, Bundoora, VIC, Australia; ^4^ Endocrinology Department, Nanfang Hospital, Southern Medical University, Guangzhou, China; ^5^ Traditional Chinese Medicine Department, Nanfang Hospital, Southern Medical University, Guangzhou, China

**Keywords:** hyperuricemia, *Zanthoxylum bungeanum*, complementary and alternative medicine, molecular docking, molecular dynamics simulation, medicine and food homologous plant

## Abstract

**Introduction:**

Hyperuricemia (HUA) is a metabolic syndrome caused by purine metabolism disorders. *Zanthoxyli Pericarpium* (ZP) is a medicinal and food homologous plant, and its ripe peel is used to treat diseases and as a spice for cooking. Some studies have shown that ZP can inhibit the formation of xanthine oxidase and reduce the production of uric acid.

**Methods:**

Through network pharmacology, ZP’s potential targets and mechanisms for HUA treatment were identified. Databases like TCMSP, UniProt, and Swiss Target Prediction were utilized for ZP’s active ingredients and targets. HUA-related targets were filtered using GeneCards, Drugbank, and Open Targets. Core targets for ZP’s HUA treatment were mapped in a PPI network and analyzed with Cytoscape. GO and KEGG pathway enrichments were conducted on intersected targets via DAVID. Molecular docking and virtual screening were performed to find optimal binding pockets, and ADMET screening assessed compound safety. Molecular dynamics simulations confirmed compound stability in binding sites.

**Results:**

We identified 81 ZP active ingredient targets, 140 HUA-related targets, and 6 drug targets, with xanthine dehydrogenase (XDH) as the top core target. Molecular docking revealed ZP’s active ingredients had strong binding to XDH. Virtual screening via Protein plus identified 48 compounds near the optimal binding pocket, with 2’-methylacetophenone, ledol, beta-sitosterol, and ethyl geranate as the most promising. Molecular dynamics simulations confirmed binding stability, suggesting ZP’s potential in HUA prevention and the need for further experimental validation.

**Conclusion:**

Our study provides foundations for exploring the mechanism of the lowering of uric acid by ZP and developing new products of ZP. The role of ZP in the diet may provide a new dietary strategy for the prevention of HUA, and more experimental studies are needed to confirm our results in the future.

## 1 Introduction

Hyperuricemia (HUA) is a metabolic syndrome caused by abnormal purine metabolism, and its diagnostic criteria are internationally defined as blood uric acid level male > 420 μmol/L (7 mg/dL), female > 357 μmol/L (6 mg/dL) (1). The prevalence of hyperuricemia is increasing globally and is on the rise in younger populations (2). Uricemia is a high-risk factor for a variety of diseases, such as gout (3), insulin resistance (4), chronic kidney disease (5), and cardiovascular disease (6), which it seriously damages public health. In the USA, an epidemiological survey showed a significant increase in the prevalence of HUA from 19.1% (1988–1994) to 21.4% (2007–2008) (7). The prevalence of HUA increases with age and is 27.2% among people aged 65 years and above, which is equivalent to 12.6 million elderly people suffering from HUA (8). Current treatments for HUA are mainly non-pharmacological and pharmacological. The non-drug treatments include improving the patient’s lifestyle and diet, limiting the intake of high-purine foods, and drinking enough water to eliminate excess uric acid from the body (9). As for the drug treatments of HUA, it is necessary to choose the appropriate drug treatment according to the cause of the patient’s increased blood uric acid, mainly through increasing uric acid excretion, inhibiting uric acid synthesis, promoting uric acid decomposition as well as assisting in lowering uric acid to achieve the effect of lowering uric acid (10). There is a high incidence of side effects associated with uric acid-lowering drugs, usually gastrointestinal discomfort, liver and kidney impairment, and increased risk of cardiovascular disease, and no new drugs with fewer side effects have been reported to be safe (11). The pathogenesis of HUA is a multifactorial and complex process, which in severe cases can lead to gout, a disease caused by excessive deposition of uric acid (12). Therefore, it is crucial to further elucidate the pathogenesis of HUA and find new therapeutic agents for HUA.

*Zanthoxyli Pericarpium* (ZP), also known as Sichuan pepper, *Zanthoxylum bungeanum*, and mountain pepper, refers to the shrubs or trees and their fruits of the Capsicum genus in the Rutaceae family (13). ZP is a very common condiment and a traditional Chinese herbal medicine with high medicinal and culinary value, widely found in Asia (14). The ripe peel of ZP is used as a condiment, to extract aromatic essential oils, and can also be used as medicine (15). Modern pharmacological studies show that ZP has anti-obesity properties (16), managing nonalcoholic fatty liver disease (NAFLD) (15)and alleviating hyperlipidemia (17). In recent years, with the development of Chinese medicine, the extraction of chemical constituents from Chinese herbal medicine has become a global research hotspot (18–20). A large number of studies have proved that ZP has pharmacological effects such as anti-inflammatory (13), antifungal and antibacterial (21), anti-aging agent and anti-tumor (14), and some studies have shown that ZP can inhibit the formation of xanthine oxidase and reduce the production of uric acid (14, 22). However, the mechanisms of action by which ZP reduces uric acid production and blood uric acid level is not clear.

In this study, we used network pharmacology, molecular docking and molecular dynamics (MD) simulation methods to investigate whether ZP mature peel has a uric acid-lowering effect and reveal its potential mechanisms of action.

## 2 Materials and methods

**Figure 1:**
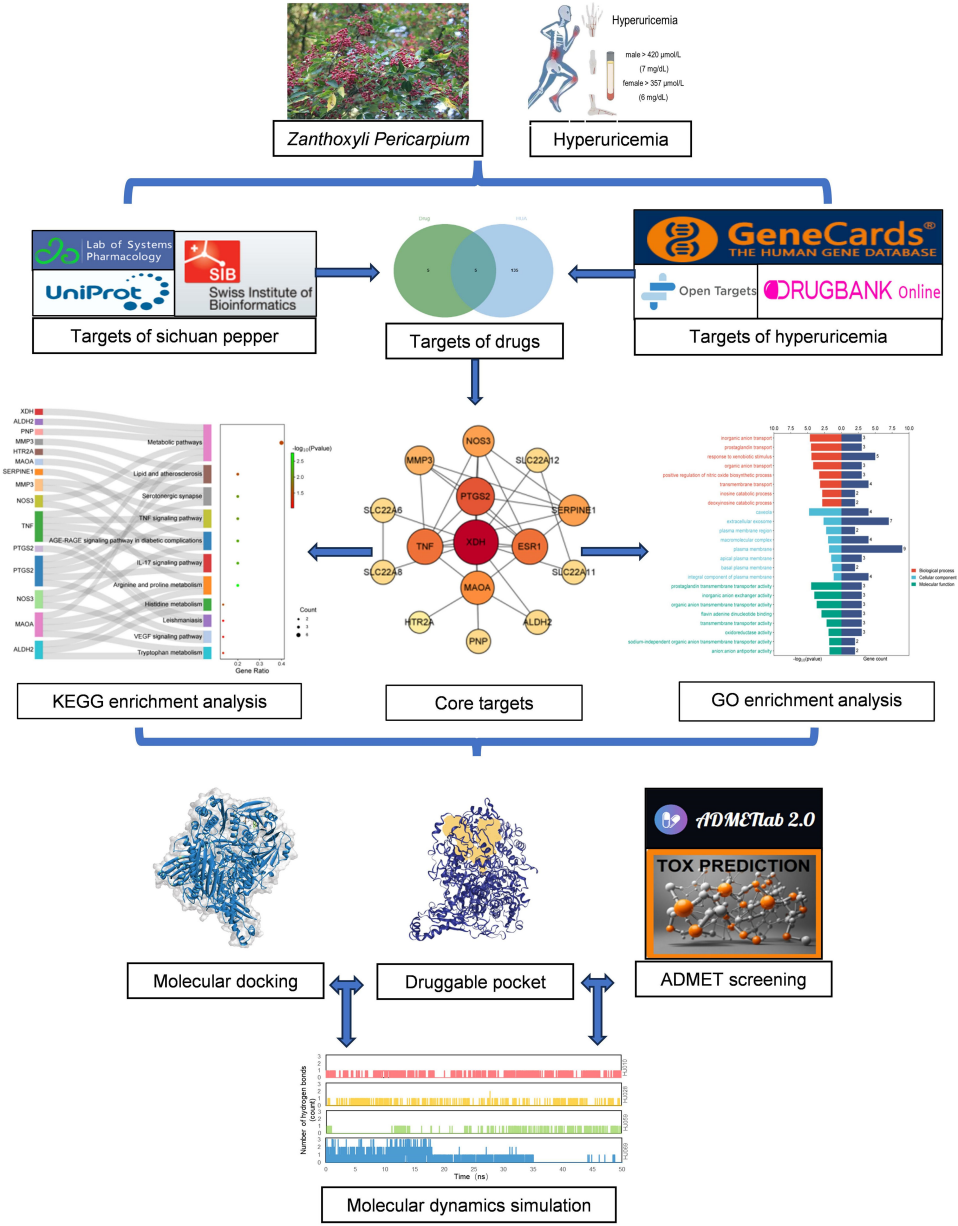
Detailed flow chart based on network pharmacology. ADMET, absorption, distribution, metabolism, excretion and toxicity; GO, Gene Ontology; KEGG, Kyoto Encyclopedia of Genes and Genomes.

In our study, the calculations were executed on the Sunway TaihuLight supercomputer, which is driven by a 12-core Chinese-developed SW26010 multi-core 64-bit RISC processor, located at the National Supercomputing Center in Wuxi, China. Additionally, we employed the SiBioLead online MD simulation platform (https://sibiolead.com), a high-performance GPU-based clustering system running on the Ubuntu operating system and NVIDIA GeForce RTX3050 Gpus. The process of our research is illustrated in the flowchart shown in **Figure 1**.

### 2.1 Identification of *Zanthoxyli Pericarpium* compounds

The compounds of ZP were derived from the Chinese Medicine Systems Pharmacology Database and Analysis Platform (TCMSP), a platform of Chinese Medicine Pharmacology (https://tcmsp-e.com/) commonly used for web-based pharmacological studies of Chinese medicine formulations (23). The platform includes all 499 Chinese herbal medicines registered in the Chinese Pharmacopoeia (2010), with a total of 12,144 chemical substances, and enables exploring their active ingredients and action targets through complex structural, omics and network system studies (24). All the information from the database developers and maintenance personnel were manually managed and updated. We collected data on their compounds using the Chinese pinyin names of Hua jiao as keywords, including molecular names, PubChem CIDs, structure files and pharmacokinetic properties (**Supplementary Table S1**). All of the data were double-checked (M.L.C and X.M.C) and the collection of any differences was discussed with a third party (H.L).

### 2.2 Collection of structures of identified ligands

The structures of all components of ZP were obtained from the TCMSP database as ligands for subsequent network interconnection analysis, docking and MD simulations. We retrieved the SMILES sequence corresponding to a PubChem CID from the PubChem database (https://pubchem.ncbi.nlm.nih.gov/), and download the 3D structure of the compound in SDF format. For compounds without 3D structures, the MOL2 structure provided by the TCMSP was used as an alternative. All structures were converted to the PDB file format using Discovery Studio Visualizer 2019.

### 2.3 Acquisition of *Zanthoxyli Pericarpium*’s potential targets for hyperuricemia

In this study, potential targets for the treatment of HUA with ZP were obtained through the following three steps (25, 26). The first step was to search and query the chemical composition of ZP from the TCMSP database, input the active ingredient into PubChem to find the SMILE structure, and the Canonical SMILES in was entered into the Swiss Target Prediction (http://swisstargetprediction.ch/) to obtain the target of the chemical composition of ZP. Swiss Target Prediction predicts the most likely small molecules of protein targets based on the similarity principle (27). We deleted duplicate targets according whose probability values were ≥ 0.05. Then, all related targets of ZP were obtained in TCMSP, and the UniProt database (https://www.uniprot.org/) was used to correct the target gene names after deletion (28, 29), and the species was limited to “Homo sapiens”. The targets corresponding to the active components of ZP were selected as those present at the intersection of the obtained targets from different databases.

The second step was to search three public disease databases, including Gene Card (https://www.genecards.org/) (30), Comparative Toxicogenomics Database (CTD) (https://ctdbase.org/) (31) and Open Targets platform (https://platform.opentargets.org/) (32, 33) to identify common disease targets associated with HUA.

In the third step, we entered the search term “hyperuricemia” into DrugBank and found five drugs, namely Allopurinol, Lesinurad, Probenecid, Rasburicase and Sulfinpyrazone. The related targets were XDH (UniProt ID P47989), SLC22A12 (UniProt ID Q96S37), SLC22A11 (UniProt ID Q9NSA0), SLC22A8 (UniProt ID Q8TCC7), SLC22A6 (UniProt ID Q4U2R8), PANX1 (UniProt ID Q96RD7), TAS2R16 (UniProt ID Q9NYV7), ABCC1 (UniProt ID P33527), ABCC2 (UniProt ID Q92887) and NR1I2(UniProt ID O75469). Intersection of HUA targets with drug targets yielded five results XDH, SLC22A12, SLC22A11, SLC22A8 and SLC22A6. In addition, considering that inhibition of purine nucleoside phosphorylase (PNP, UniProt ID P00491) to reduce uric acid production is one of the main therapeutic approaches to lower uric acid, PNP was also introduced into our study for analysis (34). We used the Open Targets database, GeneCards database and CTD database to search for potential targets of HUA by using the keyword “hyperuricemia”, and selected compounds in common across all used databases after removing duplicates.

The final cross-targets data enabled us to select a candidate for the “ZP active ingredients-HUA targets-drug targets” protein-protein interaction (PPI) map.

### 2.4 Establishment of protein-protein interaction network

To investigate the functional and regulatory interactions between proteins, we imported the active ingredient targets of ZP, the targets of HUA and drugs into the STRING database (https://string-db.org) (35)and performed PPI analysis (36). The PPI network was visualized using Cytoscape v3.8.2 to construct a network diagram of linking ZP active ingredients with HUA targets as well as drug targets. When the list of candidate gene names was entered into the database, the species was specified as Homo sapiens, and the minimum required interaction score was set to a maximum value of 0.400 (37), so that only relationships greater than this score are included in the protein network. Finally, we filtered out low-confidence interactions and hid isolated nodes to form the final PPI network (38, 39).

### 2.5 Results of Gene Ontology and Kyoto Encyclopedia of Genes and Genomes pathway enrichment analysis

To better understand the function of the screened genes, we performed functional enrichment analysis. Gene Ontology (GO) enables the analysis of gene function based on biological process (BP), cellular compound (CC), and molecular function (MF) (40, 41). The Kyoto Encyclopedia of Genes and Genomes (KEGG) enables the understanding of biological pathways associated with genes (40, 41). Both elucidated the core pathways and mechanisms of uric acid lowering by ZP. Targets were entered into the DAVID database (https://david.ncifcrf.gov/) (42, 43), identifiers were filtered based on the official gene symbol, the species was selected as homo sapiens, and then GO and KEGG pathway data were downloaded. The results in the databases were sorted according to *p*-value and visualized and designed using an online mapping tool (http://www.bioinformatics.com.cn/). In the bubble plot, the value of “-log10 (*p*-value)” is defined as the evaluation of the enrichment score, and the value of “Count” indicates the number of genes enriched in the corresponding pathway.

### 2.6 Protein structure acquisition and modification

We obtained the target names of related targets for ZP from the TCMSP database, and then searched and obtained the UniProt ID and PDBID of candidate proteins in the UniProt database (https://www.uniprot.org/), filtered by Homo sapiens species. We preferentially selected the protein with the highest resolution, and the protein with the lowest R-value is the second choice. We then downloaded the structures of the identified proteins as PDB files from the RCSB PDB protein database (https://www.rcsb.org/) (44) and examined them using the protein visualization and analysis software Visualization Molecular Dynamics. For missing fragments of proteins, we used the SWISS-MODEL server (https://swissmodel.expasy.org/) employing homology modeling to repair these structures (45). Finally, we pretreated all protein structures obtained from the RCSB database using PyRx (v0.8) (46)or modified them through homology modeling for molecular docking (47).

### 2.7 Molecular docking between *Zanthoxyli Pericarpium* compounds and candidate targets

To find the optimal binding conformation of the protein and its ligand, and to ensure the lowest binding free energy of the complex as a whole, we use molecular docking, a good tool to verify the binding affinity of the protein to the ligand (48, 49).

We investigated the interaction between ZP compounds and candidate targets using PyRx (v0.8) and AutoDock Vina (v1.1.2) (26, 50). Prior to docking, we collected the PDB files of ZP compounds and retrieved the PDB files of candidate proteins from the AlphaFold Protein Structure Database (https://alphafold.com/) (51, 52), and then converted the PDB files of ligands and targets to PDBQT format using PyRx (v0.8). Next, a docking box was drawn around the identified targets using PyRx (v0.8) with the ‘maximise’ protein outer box specification. An exhaustiveness value of 8 was used for all dockings. Finally, molecular docking was performed using AutoDock Vina (53).We added the application of CB-Dock2, a database for blind docking of proteins and ligands that integrates cavity detection, docking and homologous template fitting (54)for predicting the binding sites and affinity of ZP to candidate compounds.

In general, the lower the binding affinity, the more significant the interaction (55, 56). Based on this, we defined the binding energy value less than (greater than negative) -6 kcal/mol as possessing strong binding affinity (57, 58). The results were sorted by binding affinity score, and the lowest binding affinity score corresponded to the best binding site. Visualization was performed using Discovery Studio Visualizer 2019 (https://www.3ds.com/).

### 2.8 Screening potential candidates for treating hyperuricemia

According to the result of the docking, we calculated the average of all compounds in combination with the highest affinity targets XDH and a common drug which targets XDH as an inhibitor, Febuxostat, for further analysis of ligand and protein binding sites. The online database (https://go.drugbank.com/) includes drugs, banked PDB databases, file searches and so on. enabling determining the key contacts at protein binding sites, and then Discovery Studio Visualizer 2019 was applied to visualize the ligand-residue interactions of the herbal compounds with the two targets selected using 3D and 2D representations. For those compounds that were predicted to bind to locations in the active site pocket, we determined the total number of hydrogen bonds (H-bonds) at key active site residues. Those compounds that formed H-bonds with the key active binding site residues were uploaded to ProTox-II (https://tox-new.charite.de/protox_II/) and ADMETlab 2.0 (https://admetmesh.scbdd.com/) for predictions of the pharmacokinetic and toxicity parameters (59, 60). ADMETlab 2.0 is a powerful tool for predicting the pharmacokinetic and toxic properties of chemicals (60).

Initially in ADMETlab 2.0 screening, we filtered the molecules using parameters describing Pan Assay Interference Compounds (PAINS) (61), Lipinski’s rule of five (62, 63), human oral bioavailability (F20%) (64, 65), Volume Distribution (VD), clearance of a drug, metabolic screening (60), Hepatotoxicity(H-HT) (66), Ames (67), Rat Oral Acute Toxicity (ROA) (68), Carcinogenicity (69), and Predicted Toxicity Class (59) allowing only those ligands with drug-like properties (such as lipophilicity and solubility) to be passed through for further exploration. Then the compounds were predicted for multi-organ toxicity, graded by the ProTox-II program using SMILES of the compounds as input.

Finally, based on absorption, distribution, metabolism, excretion and toxicity (ADMET) analysis, we selected four molecules for MD simulations.

### 2.9 Molecular dynamics simulation and visualization of protein-ligand interactions

MD simulation, a method for studying protein-molecule interactions and structural changes, is increasingly being used in the fields of food development and biomedical research (70, 71). Therefore, we used MD simulation to screen compounds in ZP for the control of HUA.

SiBioLead (https://sibiolead.com) was used to perform MD simulations to predict binding stability (25). The ability of SiBioLead to perform virtual screening of large databases using high-throughput virtual screening (d-HTVS) technology allowed for the successful identification of target-specific lead molecules by screening drugs with high speed and accuracy (72). We uploaded the PDB files of *apo*- and ligand-protein complexes to the SiBioLead server and identified the ligand-protein complexes HJ010, HJ028, HJ059, and HJ069 based on the criteria of ADMET, hydrogen-bonding interactions, and binding postures, and used the OPLS all-atom force field to perform the screening using GROMACS on *apo*- and ligand-bound complexes for MD simulations (73).

To better find and visualize the crystal structure of protein structures, we used the Protein Plus server (https://proteins.plus/), a comprehensive collection of powerful web-based molecular modeling tools (74). It can efficiently predict potential binding sites, find similar binding sites for ensemble docking, and dock small molecules of interest into a binding site. We input the XDH structure into the DoGSiteScorer tool of Protein Plus and performed calculations. The results were ranked according to the drug score and subsequently, the compounds with high scores were selected for further calculations. We performed a preliminary screening using 50 ns MD simulations for the four ZP compounds and these were followed by three 200 ns MD simulations for HJ010, which had the highest score in the Protein Plus calculations, to predict the stability of the binding.

Finally, the results were derived by analyzing the trajectories using root mean square deviation of backbone Cα atoms (RMSD), root mean square fluctuation (RMSF), radium of Gyration (Rg), solvent accessible surface area (SASA) and the number of H-bonds, and the number of pairs within 0.35 nm (nPairs).

### 2.10 Cellular thermal shift assay

CETSA tests (75) the interaction of 2’ -methylacetophenone with XDH. AML12 cells were cultured in a large plate and digested by RIPA Lysis Buffer (CWBIO, CW2333S). The cell lysis solution was treated with DMSO (biosharp, BS087) or 2’-methylacetophenone (Macklin, M813230) treated in a shaker at 4°C for 2 hours with a 1:10 ratio of DMSO or 2’-methylacetophenone to protein content. The mixture was then divided into different tubes and heated at different temperatures for 3 min. After cooling, the supernatant was collected and centrifuged at 12000 rpm at 4°C for 15 min for Western bolt analysis. Cellulose membranes and primary antibodies were incubated at 4°C, and primary antibodies included rabbit monoclonal anti-XDH (1:1000, Affinity, DF8111) and Anti-rabbit IgG, HRP-linked Antibody (1:5000, Cell Signaling Technology, 7074S). Subsequently, the cellulose membrane was treated with SuperEnhanced ECL reagent (G3308, GBCBIO) for chemiluminescence detection. Luminescence signals were measured with a Tanon camera (5200S) and recorded and quantified with ImageJ.

## 3 Results

### 3.1 Active herbal compounds and potential drug targets of *Zanthoxyli Pericarpium* for hyperuricemia

**Figure 2:**
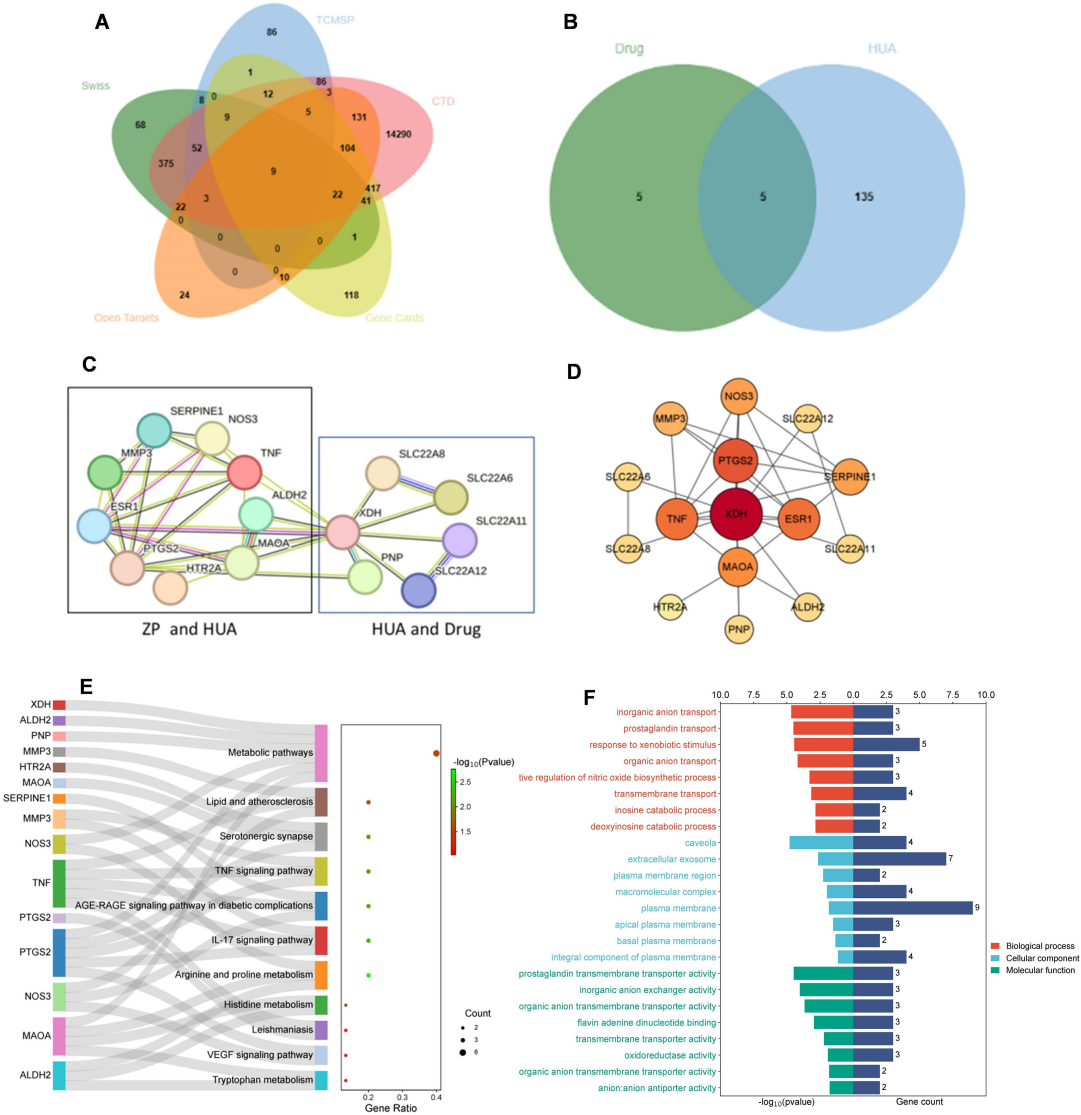
Identification and enrichment analysis of candidate targets for *Zanthoxyli Pericarpium* treating hyperuricemia. **(A)** Venn diagram showing the intersection of components of *Zanthoxyli Pericarpium* in the treatment of hyperuricemia. TCMSP, Traditional Chinese Medicine Systems Pharmacology Database and Analysis Platform; Swiss, the SwissTarget Prediction database. CTD, the CTD database; Open Targets, the Open Targets database; Gene Cards, the GeneCards database. **(B)** Venn diagram of drug intersection of targets for the treatment of hyperuricemia. Drug, the drug targets including XDH, SLC22A12, SLC22A11, SLC22A8, SLC22A6, PANX1, TAS2R16, ABCC1, ABCC2 and NR1I2. HUA, hyperuricemia. **(C)** *Zanthoxyli Pericarpium*-Disease-Drug PPI network. The nodes represent proteins, and the connecting lines between the nodes indicate interactions between two proteins, with different colors corresponding to different interaction types. Multiple connecting lines indicate multiple interactions between two proteins. **(D)** Protein-protein interaction network according to CytoNCA classification. Nodes represent proteins and node-to-node links represent associations. The color of the circular node depends on the degree of the node connected. The key genes with the highest values are marked by red nodes in the network. Higher level nodes are considered to be important hubs of the network. **(E)** KEGG enrichment analysis of the candidate targets of ZP treating hyperuricemia. The Y axis of the Sankey bubble map represents the pathway name, the X axis represents the gene ratio, the color of the points is sorted according to the *P*-value, the gene name is located on the left side of the pathway, the line represents the membership relationship, and the size of the points represents the number of genes. **(F)** The GO enrichment analysis of the candidate targets of ZP treating hyperuricemia. BP, biological process. CC, cellular component. MF, molecular function.

A total of 101 chemical components of ZP were obtained through retrieval from the TCMSP database. The obtained active components were input into PubChem to find SMILES strings, and Canonical Smiles were input into Swiss Target Prediction to obtain the target of the chemical components of ZP. Targets with probability scores ≥ 0.05 were selected (76, 77), resulting in 610 targets obtained after deleting duplicates. After deletion, the UniProt database was used to correct the gene name of the target, and the species was limited to “Homo sapiens”. A total of 274 targets corresponding to the effective active ingredients of ZP were obtained. The intersection of the ZP component targets obtained from SWISS Target Prediction platform and the corresponding targets of the TCMSP ZP component was identified, and 81 set targets were obtained. A total of 140 hyperuricemia-related targets were collected (**Figure 2A**). In the second step, we found the drug targets for HUA in DrugBank, together with PNP, and combined with the intersection of HUA targets we got five results, which were XDH, SLC22A12, SLC22A11, SLC22A8 and SLC22A6 (**Figure 2B**). Finally, the cross-targets together formed a candidate target for the “ZP-disease-drug” PPI network.

The 15 candidate targets were imported into the STRING Database for PPI analysis (**Figure 2C**). The interaction network diagram of “ZP-disease-drug” was constructed by using Cytoscape v3.8.2 to produce a visual PPI network diagram (**Figure 2D**). The top five were selected as the core genes by CytoNCA (78), and the five targets with degree centrality, betweenness centrality, and closeness centrality values greater than the average were selected as core gene targets. The top five nodes with the largest degree centrality values were XDH, PTGS2, TNF, ESR1 and MAOA, and the details are shown in **Table 1**.

**Table 1:** Classification map of candidate targets by CytoNCA.

Number	Name	Degree Centrality	Betweenness Centrality	Closeness Centrality
1	XDH	11	103.14286	0.8235294
2	PTGS2	8	20.238094	0.7
3	TNF	7	11.238095	0.6666667
4	ESR1	7	11.238095	0.6666667
5	MAOA	6	30.857143	0.6363636
6	NOS3	5	2.7857144	0.5833333
7	SERPINE1	5	0.5	0.4827586
8	MMP3	4	0	0.46666667
9	SLC22A8	2	0	0.4827586
10	SLC22A6	2	0	0.4827586
11	SLC22A12	2	0	0.4827586
12	SLC22A11	2	0	0.4827586
13	PNP	2	0	0.5185185
14	ALDH2	2	0	0.5
15	HTR2A	1	0	0.4

The PPI network graph (**Figure 2C**) for ZP-HUA-drug contained 33 edges and 15 nodes, with an average node degree of 4.4 and a local clustering enrichment coefficient of 0.834. The *p*-value for PPI enrichment was small and statistically significant at < 1.0×10^-12^. This implied that there was a high degree of gene-gene interactions, and such enrichment suggested that the genes, as a group, were at least partially connected biologically.

### 3.2 *Zanthoxyli Pericarpium*’s involvement in multiple signaling pathways

To clarify the interaction between the drug and target, GO and KEGG enrichment analyses were performed. A total of 48 BP, 8 CC and 11 MF items were obtained from the DAVID database, and 11 KEGG pathways were enriched, indicating the multi-pathway characteristics of ZP control of uric acid (**Figure 2E**).

According to the GO enrichment analysis results in the DAVID database, the genes were enriched into different GO terms, and the top eight GO terms in the three categories were selected to construct connections within the signal network according to the order from smallest to largest *p*-value (**Figure 2F**). The *x*-axis in the bar represented log_10_ (*p*-value) and the number of targets, while the *y*-axis represented the GO term. The top eight BP included response to inorganic anion transport, prostaglandin transport, response to xenobiotic stimulus, organic anion transport, positive regulation of nitric oxide biosynthetic process, transmembrane transport, inosine catabolic process and deoxyinosine catabolic process. Through CC analysis, caveola, extracellular exosome, plasma membrane region, macromolecular complex, plasma membrane, apical plasma membrane, basal plasma membrane and integral component of plasma membrane were listed as the top eight categories. In addition, top-ranking signaling pathways mainly included arginine and proline metabolism, IL-17 signaling pathway, AGE-RAGE signaling pathway in diabetic complications, TNF signaling pathway, serotonergic synapse, histidine metabolism, lipid and atherosclerosis, metabolic pathways, tryptophan metabolism, VEGF signaling pathway and leishmaniasis.

### 3.3 Overall evaluation of *Zanthoxyli Pericarpium* compounds docking with hyperuricemia proteins

Based on the PPI network results, 15 therapeutic targets for HUA were identified and selected for molecular docking (**Table 2**). Semi-flexible docking was first performed with the target protein receptor structure and 101 ZP compounds, which yielded a total of 1515 molecular docking results. The results showed that 54 compounds (53.47%) had an average binding energy of lower than -6 kcal/mol, and the binding energies were distributed between -2.087 kcal/mol and -11.246 kcal/mol. Among them, the top ten target protein receptors with the highest average binding scores were XDH, MMP3, MAOA, SLC22A8, SLC22A6, NOS3, HTR2A, PTGS2, SLC22A12 and SLC22A11. For detailed information on the interconnections, see **Supplementary Table S2**.

**Table 2:** Details of the 15 candidate targets of *Zanthoxyli Pericarpium* treating hyperuricemia.

Serial number	UniProt ID	Target name	Total binding score (kcal/mol)	Average binding affinity(kcal/mol)
HH10	P47989	XDH	-661.98	-6.554257426
HH08	P08254	MMP3	-646.036	-6.39639604
HH01	P21397	MAOA	-644.601	-6.382188119
HH15	Q8TCC7	SLC22A8	-635.634	-6.293405941
HH14	Q4U2R8	SLC22A6	-623.511	-6.173376238
HH02	P29474	NOS3	-623.058	-6.168891089
HH09	P28223	HTR2A	-621.62	-6.154653465
HH05	P35354	PTGS2	-620.493	-6.14349505
HH12	Q96S37	SLC22A12	-613.831	-6.077534653
HH13	Q9NSA0	SLC22A11	-597.844	-5.919247525
HH11	P00491	PNP	-592.523	-5.866564356
HH03	P05091	ALDH2	-590.584	-5.847366337
HH06	P03372	ESR1	-570.203	-5.645574257
HH07	P05121	SERPINE1	-565.908	-5.603049505
HH04	P01375	TNF	-512.298	-5.072257426

ALDH2, aldehyde dehydrogenase, mitochondrial; ESR1, estrogen receptor 1; HTR2A, 5-hydroxytryptamine receptor 2A; MAOA, monoamine oxidase type A; MMP3, matrix metalloproteinase-3; NOS3, Nitric oxide synthase 3; PNP, purine nucleoside phosphorylase; PTGS2, prostaglandin G/H synthase 2; SERPINE1, plasminogen activator inhibitor 1; SLC22A11, solute carrier family 22 member 11; SLC22A12, solute carrier family 22 member 12; SLC22A6, solute carrier family 22 member 6; SLC22A8, solute carrier family 22 member 8; TNF, tumor necrosis factor; XDH, xanthine dehydrogenase.

**Figure 3:**
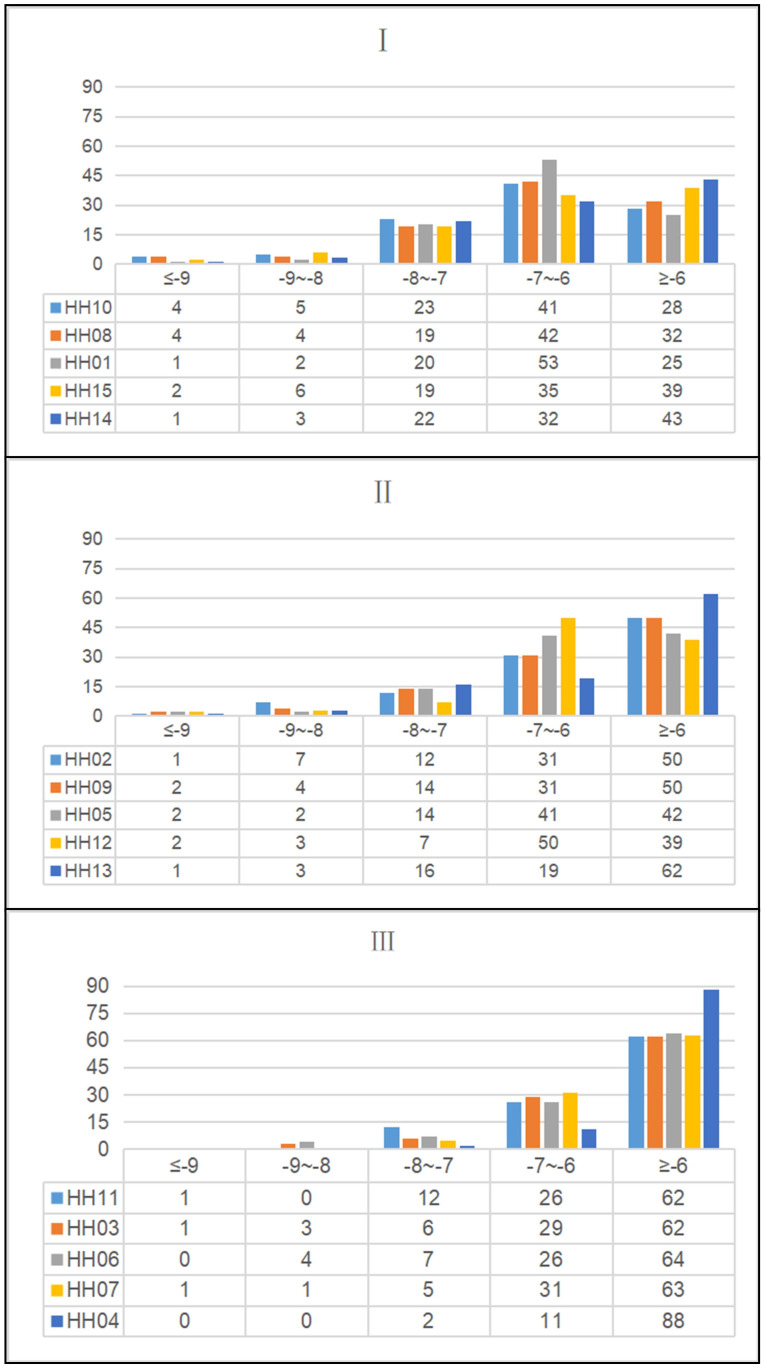
Histogram and data table of molecular docking results in the treatment of hyperuricemia with *Zanthoxyli Pericarpium*. The color of the histogram represents ZP compounds, and the data table shows the number of molecular docking score intervals.

We further classified the average binding energies of the docking results into three clusters (**Figure 3**). In the first cluster, HH10, HH08, HH01, HH15 and HH14 were the groups with the most negative total binding energies. Among them, the target protein XDH had the highest number of combinations with binding energies of less than -6 kcal/mol with ZP compounds, indicating that XDH has a good binding affinity for ZP and can play a role in the control of uric acid. Secondly, the binding energies of the second cluster, composed of HH02, HH09, HH05, HH12 and HH13, were lower than that of the first class, and the combinations of binding energies of this group of target proteins were mainly greater than -7 kcal/mol. Finally, the low binding energy target proteins HH11, HH03, HH06, HH07, and HH04 had more compounds with binding energy greater than -6 kcal/mol than the rest of the classification group and were therefore classified as one cluster.

### 3.4 Visualization of binding modes adopted by tight binding ligands predicted to interact with *Zanthoxyli Pericarpium* and XDH

**Figure 4:**
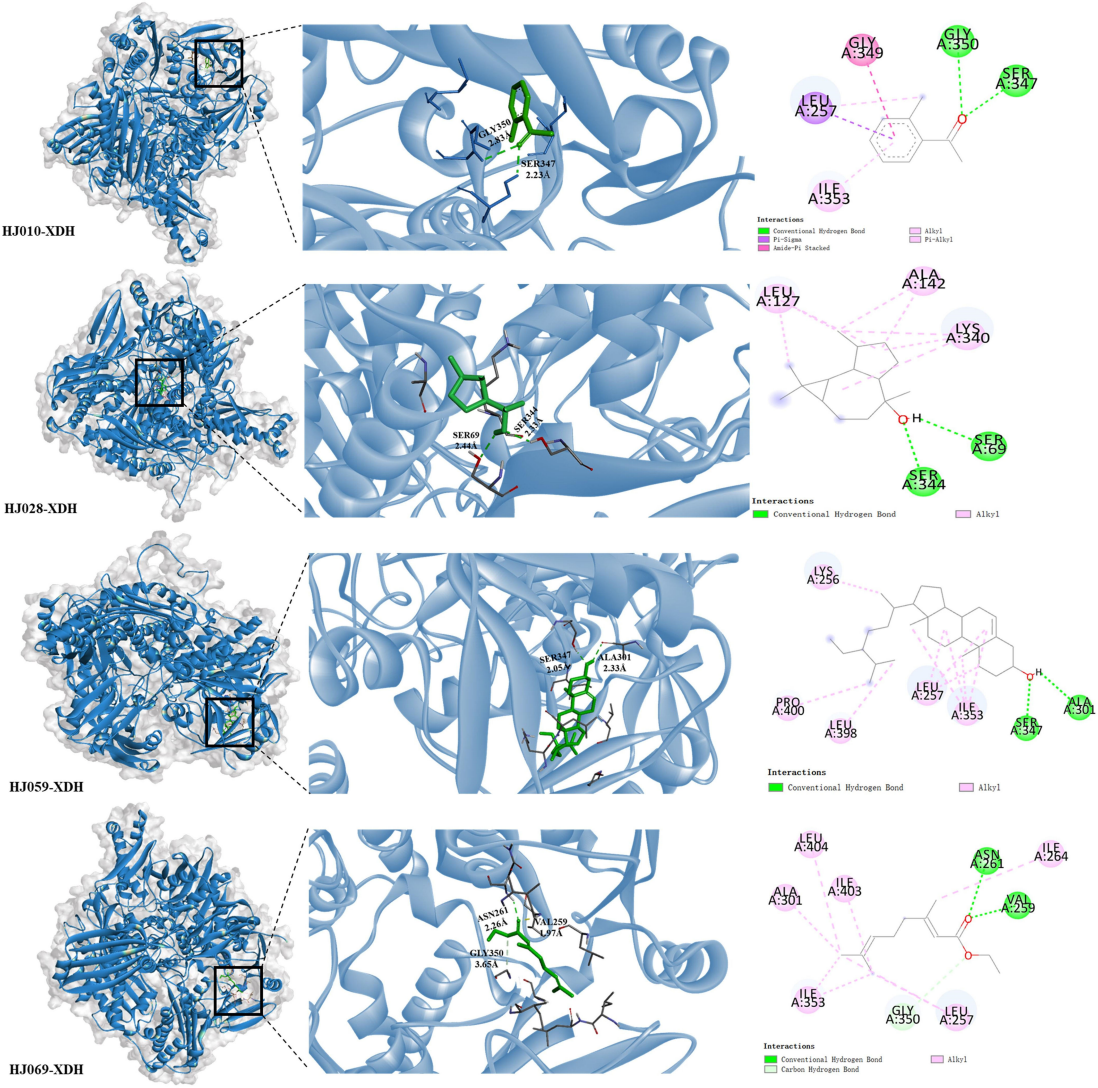
Structural diagram of molecular docking of candidate compounds. **(A)** HJ010-XDH, **(B)** HJ028-XDH, **(C)** HJ059-XDH and **(D)** HJ069-XDH.

The XDH inhibitor febuxostat reduces the uric acid formation and lowers blood uric acid levels by tight binding to the active site of the XO molybdenum pterin center, which keeps the molybdenum cofactor in its oxidized or reduced state in isolation, and thus exerts an inhibitory effect on the aggregation of both XO and substrate (79). Febuxostat was predicted to dock with XDH and according to our previous research (25) and related literature (80), the conventional binding site included the residues Asn769, Glu803, Arg881 and Thr1011. The compounds of ZP were docked with XDH (**Figure 4**), and H-bonds were identified in a number of binding sites, which may be involved in the biological action of uric acid lowering (**Table 3**).

**Table 3:** Screening of candidate compounds for treating hyperuricemia of *Zanthoxyli Pericarpium*.

Component	Hydrogen bonding at inactive sites	Pockets of druggability	Average binding energy<-6 Kcal/mol	Candidate compound
HH010	Ser347、Gly350	Top1	-6.301	HJ010
HH016	Gly260、Asn261、Thr262、Glu263	Top1	-7.062	HJ016
HH022	Leu404	Top1	-8	HJ022
HH026	Leu257	Top1	-7.049	HJ026
HH027	Ser69、Ser344	Top1	-6.991	HJ027
HH028	Ser69、Ser344	Top1	-7.434	HJ028
HH030	Lys249、Leu404	Top1	-7.71	HJ030
HH037	Ser347	Top1	-6.521	HJ037
HH047	Ala301	Top1	-6.455	HJ047
HH051	Gly260	Top1	-7.398	HJ051
HH053	Asn261、Thr262	Top1	-8.985	HJ053
HH059	Ala301、Ser347	Top1	-9.52	HJ059
HH064	Gln62、Ser69、Ser344	Top1	-9.103	HJ064
HH069	Val259、Asn261	Top1	-6.763	HJ069
HH074	Pro253、Lys256、Asn261、Ile264、Arg394、Pro400	Top1	-11.246	HJ074
HH077	Thr2Gln62、Glu263	Top1	-8.692	HJ077
HH080	Asn261、Thr2Gln62、Glu263	Top1	-6.508	HJ080
HH100	Lys249	Top1	-6.583	HJ100
HH101	Val259、Ile264	Top1	-9.638	HJ101

The Composition column represents the components of *Zanthoxyli Pericarpium* docked with xanthine dehydrogenase (XDH). Hydrogen bonding at inactive sites represents the inactive sites where XDH and ZP docking can produce hydrogen bonding. The Pockets of druggability column represents whether the compound came from the druggability pocket of Top1. The mean binding energy of the compounds are lower than -6 Kcal/mol, and the candidate compounds represent the candidate compounds for the treatment of hyperuricemia of *Zanthoxyli Pericarpium*.

We imported the structures of XDH and ZP into the Protein Plus server in PDB format. Then we used DoGSiteScorer to predict 49 potential drug-forming pockets (**Supplementary Table S3**). The closer the drug score is to 1, the more the drug-like properties are, and the more the region can be a drug target (74). Cavities of different shapes and structures in the protein structure are defined as pockets and ligand binding occurs in adjacent cavities (81). Pharmaceutical development distinguishes between drug and non-drug pockets mainly by pocket size, shape complexity and hydrophobicity, with drug development favoring larger, more complex and hydrophilic pockets (82).

**Figure 5:**
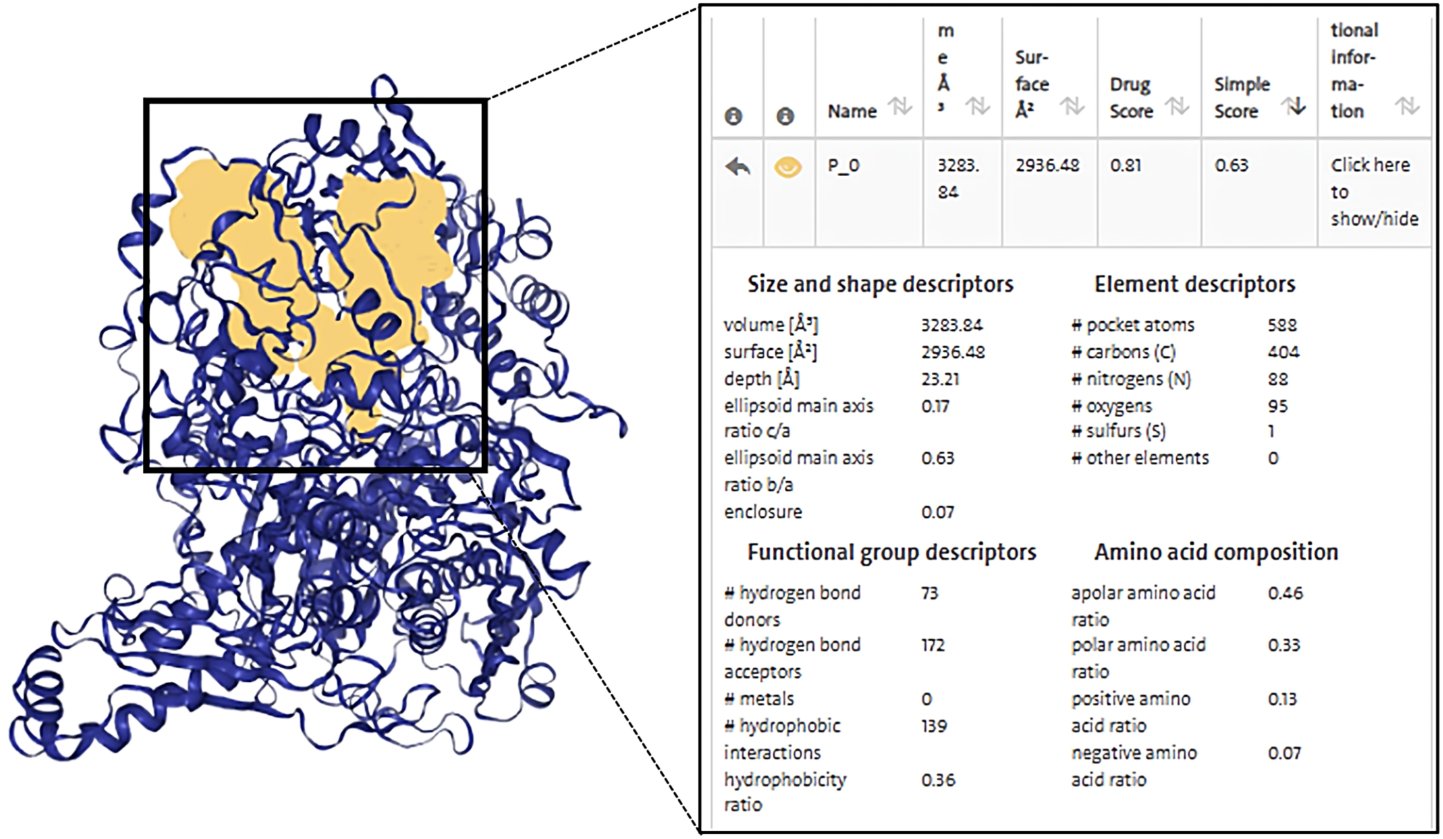
Diagram of the structure of a druggable pocket and DoGSiteScorer Score content. The score contents are sorted by the highest simple score and the yellow area in the box shows the structure of the druggable pocket 0, which has the highest simple score.

Based on this, first, we ranked the simple score, which is the result of a linear combination of simple distinguishing pocket features capable of distinguishing between drug and non-drug pockets (82), showing that Pocket 0 has the highest simple score of 0.63, a drug score of 0.81, volume of 3283.84 Å^3^, surface area of 2936.48 Å^2^, and depth of 23.21 Å. The DoGSiteScorer score is greater than 0.5 and is considered a druggable pocket (**Figure 5**, **Supplementary Table S3**).

The second step was to consider the volume and depth of the pocket. The true active site often overlaps with the largest protein pocket, and the algorithm predicts binding performance by calculating pocket volume (82). It was shown that the average volume of the druggable pockets is 900 Å^3^ and the druggable pockets are deeper, with an average depth value of 21 Å (82), while the volume of pocket 0 was 3283.84 Å^3^ and had a depth up to 23.21 Å, which were much greater than the average, suggestive of the possibility that the ZP compound binds to XDH at pocket 0 and exerts biological activity.

Finally, the druggability of the pockets was considered in terms of physicochemical properties. The quotient of gps_se_h, meaning the relative number of hull grid points, is used to describe enclosure, with the quotient being higher if drug-free pockets are more solvent-exposed, indicating a higher solvent exposure (low probability of drug binding) for pockets with hull grid point ratio of 0.17, compared to 0.08 for drug-containing pockets (82). Pocket 0 had an enclosure of 0.07, a pocket with drug-forming potential. Drug binding was dominated by hydrogen bonding and ionic interactions. There were 73 hydrogen bond donors and 172 hydrogen bond acceptors, 139 hydrophobic structures, and 7 valine residues at this pocket. Docking indicated that there are 48 herbal compounds in the vicinity of pocket 0. Screening for compounds that could produce hydrogen bonds in the active site, and based on the results of ZP docking with XDH molecules, we also filtered for compounds with binding energies less than or equal to -6 kcal/mol, resulting in a list of 19 potential piperine compounds (**Supplementary Tables S3**, **S4**).

### 3.5 Pharmacokinetic screening of active ingredients in *Zanthoxyli Pericarpium* for the amelioration of hyperuricemia

To obtain compounds of ZP that have the potential to become drugs for development, we entered the SMILES structures of 101 components of ZP into the ADMETlab 2.0 website (https://admetmesh.scbdd.com/) for prediction. We defined the sequential principles of pharmacokinetic screening, firstly screening out monomers with a PAINS score of one, and secondly removing compounds which show a violation of any of the Lipinski Rule of Five. Thirdly, compounds with a probability of oral bioavailability of 20%, volume of drug distribution, hepatotoxicity, mutagenicity, acute oral toxicity in rats, and carcinogenicity were screened out. Based on the simulation of drug metabolism in the human body, ingredients with an oral bioavailability greater than 0.7 in humans and a drug clearance of less than 5 are difficult to metabolize in the body (83) and are therefore excluded.

ZP, in addition to being used as an herbal medicine, is also an everyday spice found in many dishes. It is therefore important to make sure that the compounds of the drug derived from ZP do not exceed the oral toxicity dose. The web server ProTox-II uses computer simulation methods to predict the toxicity of various toxicity endpoints (59). A predicted toxicity rating IV or higher was defined as the safe range. These compounds may exhibit similar function to XDH inhibitors and may produce better uric acid-lowering effects (**Table 4**).

**Table 4:** ADMET screening of candidate compounds from *Zanthoxyli Pericarpium*.

Candidate compound	Medicinal Chemistry		Absorption	Distribution	Metabolism		Excretion	Toxicity					Compounds with drug potential
	PAINS	Lipinski Rule	F20%	VD	CYP 1A2/2C19/2C9/2D6/3A4 inhibitor	CYP 1A2/2C19/2C9/2D6/3A4 substrate	CL	H-HT	AMES	ROA	Carcinogenicity	Predicted Toxicity Class	
HJ010	0	Accepted	0.004	0.818	0.4396	0.5876	6.597	0.052	0.104	0.051	0.507	4	HJ010
HJ016	0	Accepted	0.927	2.704	0.2954	0.2956	9.707	0.917	0.012	0.014	0.3	5	
HJ022	0	Accepted	0.002	0.435	0.7356	0.6882	11.219	0.134	0.459	0.734	0.76	4	
HJ026	0	Accepted	0.002	1.575	0.2418	0.4796	9.232	0.343	0.901	0.571	0.815	5	
HJ027	0	Accepted	0.295	0.908	0.5788	0.7054	4.503	0.098	0.806	0.1	0.38	3	
HJ028	0	Accepted	0.017	1.179	0.1264	0.6062	18.537	0.261	0.008	0.436	0.029	4	HJ028
HJ030	0	Accepted	0.117	0.618	0.6408	0.6278	12.128	0.31	0.061	0.386	0.863	2	
HJ037	0	Accepted	0.027	1.092	0.623	0.8436	11.466	0.067	0.044	0.029	0.798	4	
HJ047	0	Accepted	0.732	0.833	0.6136	0.7534	14.042	0.036	0.066	0.121	0.814	4	
HJ051	0	Accepted	0.002	0.413	0.7162	0.8448	8.747	0.162	0.635	0.739	0.667	4	
HJ053	0	Accepted	0.949	0.657	0.7094	0.5718	7.066	0.066	0.508	0.035	0.055	5	
HJ059	0	Accepted	0.01	1.963	0.0842	0.5912	16.686	0.16	0.026	0.018	0.047	4	HJ059
HJ064	1	Rejected	0.477	0.904	0.0428	0.1186	5.369	0.144	0.809	0.073	0.043	5	
HJ069	0	Accepted	0.351	2.46	0.6638	0.5282	8.496	0.42	0.003	0.009	0.108	5	HJ069
HJ074	0	Rejected	0.045	0.61	0.009	0.1274	1.387	0.062	0.743	0.021	0.715	5	
HJ077	0	Accepted	0.304	0.925	0.6	0.673	13.319	0.795	0.031	0.036	0.584	4	
HJ080	0	Accepted	0.116	0.74	0.1642	0.2648	2.573	0.018	0.004	0.016	0.084	2	
HJ100	0	Accepted	0.004	1.177	0.3906	0.4748	2.727	0.019	0.023	0.024	0.248	4	
HJ101	1	Accepted	0.93	0.579	0.4706	0.21	8.284	0.1	0.657	0.065	0.05	3	

PAINS, Pan Assay Interference Compounds, showed that 1 of the pain compounds were screened for false positive results or suspected compounds. Lipinski Rule, if more than 2 items of Lipinski Rule were violated, rejected was shown. The probability of oral bioavailability of 20% is greater than 0.7, indicating good bioutilization effect. VD, Volume Distribution, is an important parameter that describes the distribution of drugs in the body, with a value between 0.04 and 20. The output value in metabolic screening represented the probability of being a substrate/inhibitor and ranged from 0 to 1. CL, clearance of a drug, is an important pharmacokinetic parameter, which defines the volume of drug distribution, half-life, and thus the frequency of drug administration. A drug clearance of <5 ml/min/kg means low clearance. H-HT, drug-induced liver injury, which output value represents the probability of causing injury. The Ames test for mutagenicity, which effect has a close relationship with the carcinogenicity. Rat Oral Acute Toxicity is one of the most important tasks for the safety evaluation of drug candidates. An output value greater than 0.7 for H-HT, Ames, ROA and Carcinogenicity indicates that the compound is likely to be carcinogenic or toxic. The predicted toxicity grade was divided into five levels, Class I: fatal if swallowed (LD_50_ ≤ 5); Class II: fatal if swallowed (5 < LD_50_ ≤ 50); Class III: toxic if swallowed (50 < LD_50_ ≤ 300); Class IV: harmful if swallowed (300 < LD_50_ ≤ 2000); Class V: may be harmful if swallowed (2000 < LD_50_ ≤ 5000); Class VI: non-toxic (LD_50_ > 5000).

Based on the above conditions, we identified the following four herbal compounds as possessing likely drug-forming potential, namely HJ010, HJ028, HJ059, and HJ069, for the next step of experiments as well as MD simulations.

### 3.6 Molecular dynamics simulations to verify the stability of XDH binding to *Zanthoxyli Pericarpium*’s ingredients

**Figure 6:**
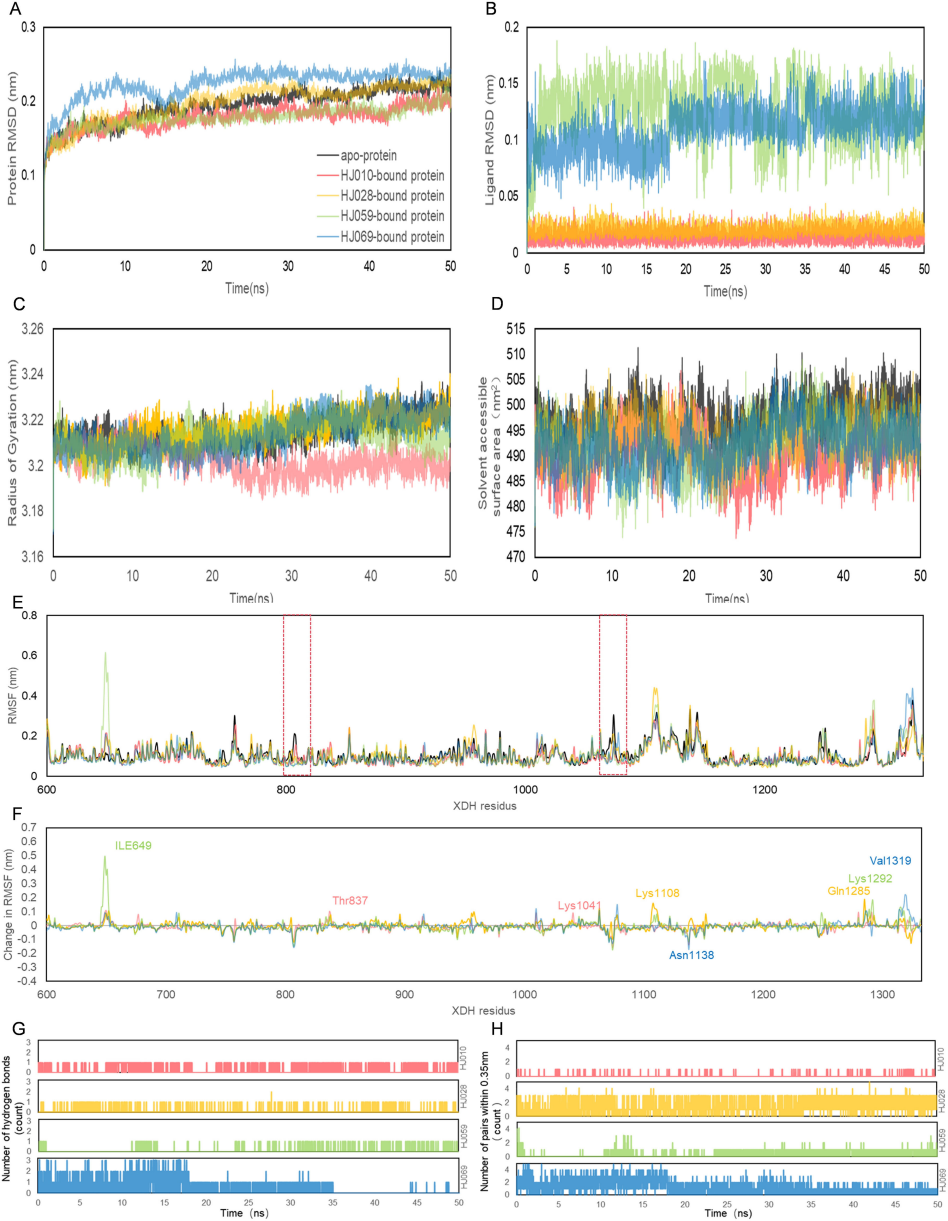
Structural analyses of the *apo-*, HJ010-, HJ028-, HJ059- and HJ069-bound XDH based on molecular dynamics simulation. The four inhibitors’ values of selected parameters are illustrated as follows, including protein **(A)** RMSD, **(B)** ligand RMSD, **(C)** Rg, **(D)** SASA, **(E)** RMSF, **(F)** change in RMSF, **(G)** the number of hydrogen bonds and **(H)** the number of pairs within 0.35 mm.

To verify the stability of the docked structures, we selected four ZP compounds: HJ010, HJ028, HJ059, and HJ069 for MD simulation with XDH and analyzed the docking results by interpreting six parameters (**Figure 6**, **Table 5**, **Supplementary Table S5**).

**Table 5:** Structural analyses of stabilized trajectories following equilibration at 10 ns.

Analysis	XDH-apo	XDH -HJ010	XDH -HJ028	XDH -HJ059	XDH-HJ069
Protein RMSD (nm)	0.203 ± 0.014	0.184 ± 0.011	0.208 ± 0.015	0.184± 0.009	0.230± 0.012
Ligand RMSD (nm)	N/A	0.015 ± 0.006	0.021 ± 0.005	0.129 ± 0.025	0.114 ± 0.018
Radius of gyration (nm)	3.216 ± 0.006	3.201 ± 0.006	3.218 ± 0.006	3.214 ± 0.006	3.215 ± 0.008
Solvent accessible surface area (nm^2^)	497.8 ± 3.969	490.119 ± 4.446	494.235 ± 3.906	492.086 ± 5.289	492.435 ± 4.407
RMSF (nm)	0.113 ± 0.056	0.104 ± 0.050	0.111 ± 0.073	0.112 ± 0.065	0.111 ± 0.066
Number of hydrogen bonds	N/A	1069 ± 0.443	204 ± 0.221	322 ± 0.272	1358 ± 0.626
Number of pairs within 0.35 nm	N/A	111 ± 0.164	6158 ± 1.002	1695 ± 0.521	4170± 0.988

N/A, not applicable; RMSD, root mean square deviation of backbone Ca atoms; RMSF, root mean square fluctuation; XDH, xanthine dehydrogenase; HJ010, 2’-methylacetophenone; HJ028, ledol; HJ059, beta-sitosterol;HJ069, ethyl geranate.

RMSD calculations for the ligand-free and ligand-bound proteins are depicted in **Figures 6A, B**. All systems were observed to be relatively stable for the entire 50 ns, with no major displacement beyond 0.26 nm. After about 15 ns, all systems showed relatively small fluctuations indicating that all systems achieved equilibration. Interestingly, the RMSD of the proteins bound by HJ010 and HJ059 was lower than that of the remaining two proteins. The binding proteins of HJ028 and HJ069 had a higher average RMSD value, whereas the binding energy of the HJ059 ligand was higher. Although the trend and magnitude of the RMSD of the protein bound to HJ059 were very similar to those of *apo*-protein, the RMSD of the ligand itself (HJ059) was relatively high. This indicated that HJ010 and HJ028 have greater stability in their initial structure at the XDH active site than other ligands.

Protein conformation changes measured by Rg over time are shown in **Figure 6C**, while SASA changes are displayed in **Figure 6D**. Except for HJ028-bound systems, all the rest had no major changes in protein conformation, and the standard deviation was relatively narrow, less than 0.01 nm, indicating that the conformation of proteins showed high stability and compactness. HJ010-bound had the lowest average value of Rg, 3.202 nm, and stabilized to this value after 15 ns of simulation. In agreement with the Rg findings, the SASA values of the HJ010-binding protein were also significantly decreased. This decrease began at an earlier time frame, around 10-20 ns, and possibly indicating that the protein conformation was destabilized upon binding to HJ010.

The RMSF and change in RMSF curves reflected fluctuations in the amino acid residues of the proteins, as shown in **Figures 6E, F**. A general examination of the site-specific residues of the ligand-bound and ligand-free structures indicated that they showed similar flexibility. Most of the peaks are located around the loop region and are highly overlapping with each other. The results for *apo* XDH compared to the ligand-bound XDH indicated that amino acids Val808 and Asn1074 of XDH had greater residue flexibility than the other regions. The significant suppression of residue fluctuations observed in these regions due to binding with HJ010 (Thr837, Lys1041), HJ028 (Lys1108, Gln1285), HJ059 (Ile649, Lys1292), and HJ069 (Asn1138, Val1319) in all ligand-bound structures suggested that this region may be important for ligand-binding and the subsequent stability of XDH protein structures.

The number of H-bonds and pairs within 0.35 nm are presented in **Figures 6G, H**, respectively. The number of H-bonds generated stabilized after 50ns, indicating that HJ010 produced the most persistent H-bonds, followed by HJ069. For the HJ069 ligand, as the system approached equilibrium, it gained polar contacts with XDH residues and maintained a high number of contacts until approximately 35 ns, before losing some contacts in the second half of the simulation. This observation could be attributed to a conformational change in the ligand and is consistent with the ligand RMSD. HJ059 stably formed a hydrogen bond most of the time. Although no polar contact was detected between the HJ028 and XDH structures, other major hydrophobic interactions may be responsible for the binding stability of HJ028, despite significantly weaker binding compared to ligands with hydrogen bonding capability.

### 3.7 Molecular dynamics simulation of 2’-methylacetophenone complex to XDH for 200 ns

**Figure 7:**
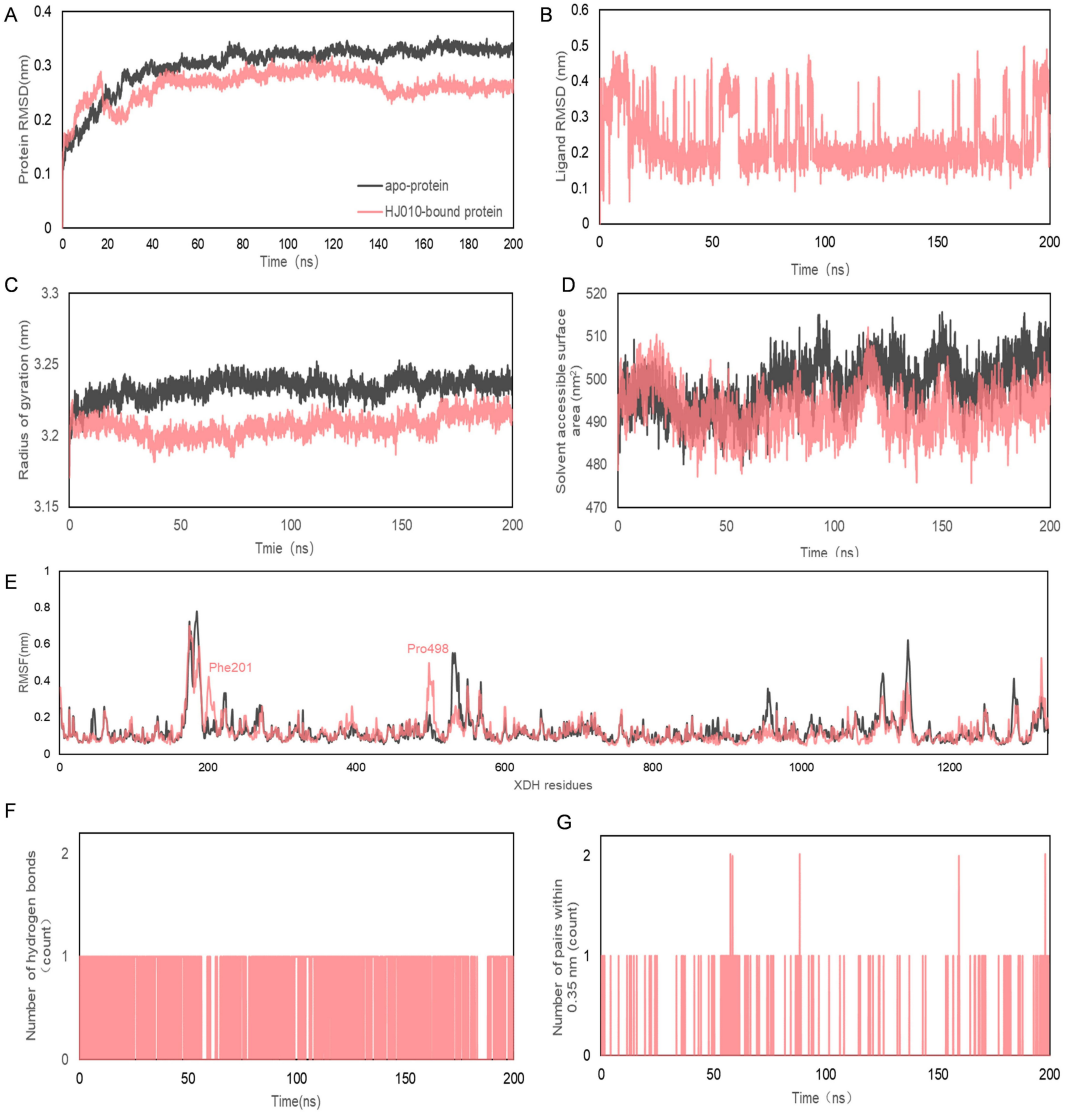
Molecular dynamics simulation of 200 ns trajectories for *apo*- and HJ010- XDH complex. **(A, B)** RMSD **(C)** Radius of gyration for *apo*- and HJ010-XDH complex. **(D)** Solvent accessible surface area. **(E)** RMSF diagrams obtained from molecular dynamics simulation of *apo*- and HJ010- XDH complex. **(F, G)** Hydrogen bonds between ligands and proteins during molecular dynamics simulations.

After an initial screening of 50 ns MD simulations, we concluded that the HJ010 structure, 2’-methylacetophenone, was more tightly and stably bound to XDH. Therefore, we performed 200 ns MD simulations in triplicate and integrated this data together with the shorter trajectories initially obtained by the SiBioLead platform to better simulate the trajectory of the structure’s action in binding to XDH *in vivo* (**Figure 7**, **Table 6**, **Supplementary Table S6**), and to confirm the stability of the ligand under a longer simulation timescale.

**Table 6:** Molecular dynamics simulations of 200 ns XDH with 2’-methylacetophenone in triplicate.

Analysis	XDH-*apo*	XDH -HJ010
Protein RMSD (nm)	0.303 ± 0.044	0.262 ± 0.028
Ligand RMSD (nm)	N/A	0.237 ± 0.082
Radius of gyration (nm)	3.234 ± 0.007	3.207 ± 0.007
Solvent accessible surface area (nm^2^)	498.635 ± 5.854	492.777 ± 5.232
RMSF (nm)	0.130 ± 0.087	0.127 ± 0.077
Number of hydrogen bonds	N/A	3527 ± 0.456
Number of pairs within 0.35 nm	N/A	302 ± 0.242

N/A, not applicable; HJ010, 2’-methylacetophenone.

The RMSD curve reflected the fluctuation of protein conformation, as shown in **Figures 7A, B**, indicating that the RMSD had some fluctuation in the early stage of the trajectory. However, after about 30 ns the fluctuation was less than that of *apo* XDH and stabilized. This suggested that there was no major change in the conformation of 2’-methylacetophenone after binding to XDH, and the combination of the two resulted in a relatively stable complex.

Rg and SASA were often used as a measure of protein structural compactness and changes in protein exposure to solvents (84) (**Figures 7C, D**). An increase in the Rg value implied a decrease in the structural compactness of the protein, which indicated greater flexibility and less stability (85). The Rg of 2’-methylacetophenone with XDH was more stable after 6.32 ns. The SASA values of *apo*-protein (black) and HJ010-bound protein (pink) ranged from 475.89 to 515.714 nm^2^, suggesting that the folding pattern of the proteins did not change significantly due to binding to the ligand and therefore were likely to remain stable throughout the simulation.

The average RMSF values between the *apo-* and HJ010-bound structures exhibited small differences around two regions surrounded by peaks at residues Phe201 and Pro498, as shown by the red dashed boxes in **Figure 7E**. Intermolecular hydrogen bonding was one of the most important interactions between proteins and ligands and played a key role in the stability of the complexes (86). The distribution and number of hydrogen bonds between ligands and proteins during MD simulations are shown in **Figure 7F**. HJ010 maintained a large number of hydrogen bonds during the 200 ns simulation. MD simulation studies showed that the ligand-receptor complex of HJ010 with XDH was stable. 

### 3.8 2'-methylacetophenone and XDH form a stable complex

**Figure 8:**
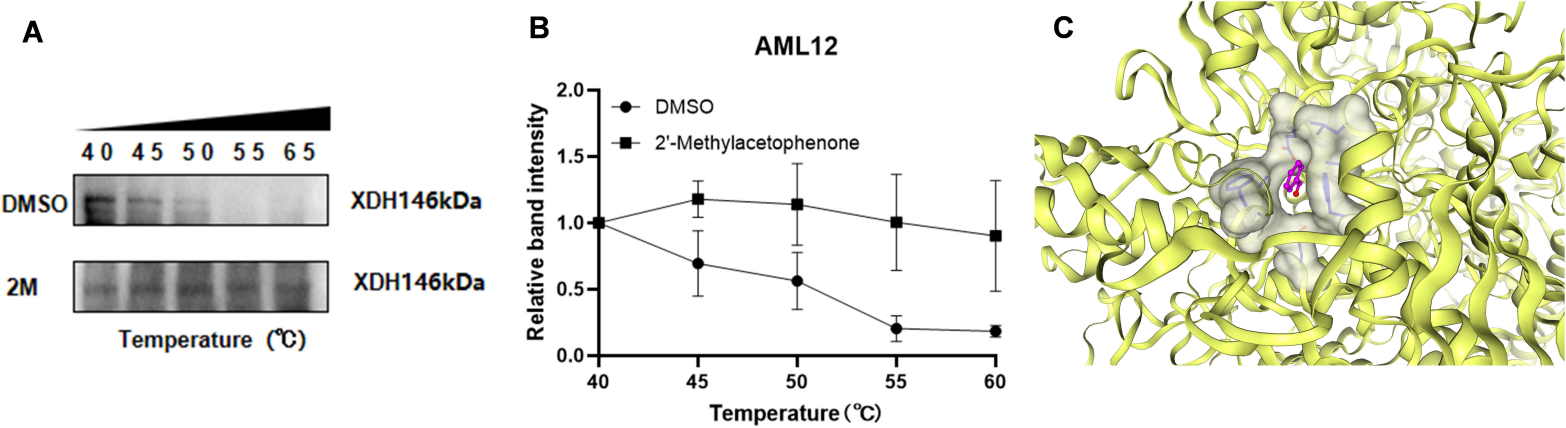
CESTA for interaction between 2’-methylacetophenone and XDH. **(A)** A representative graph of CETSA and **(B)** the density of the bands were measured and shown as a line chart. Values are expressed as mean ±SEM (n = 3). **(C)** 2’-methylacetophenone and XDH molecular docking of the most fit predictive pocket schematic, via CB-Dcok2.

To verify the binding stability of 2'-methylacetophenone and XDH, we have verified the stability of the combination of HJ010 and XDH through CETSA (**Figures 8A, B**). CETSA (75) is an experiment that measures how efficiently a drug binds to a target protein inside a cell, based on the principle that the target protein usually becomes stable when it binds to the drug molecule. That is, with the increase of temperature, the protein will degrade. When the protein binds to the drug, at the same temperature, The amount of non-degraded protein will increase. With the increase of temperature, the expression of XDH after the intervention of HJ010 was more and the binding was more stable, compared with DMSO group. In addition to this, we add the application of CB-Dock2, a database for blind docking between proteins and ligands, integrating cavity detection, docking and homologous template fitting (54). Five blind grafting results proved that the combination of HJ010 and XDH was stable (**Supplementary Table S7**). One of the most suitable binding pockets and a 2015 article "Aromatic aldehydes at the active site of aldehyde oxidoreductase from desulfovibrio gigas: reactivity and molecular details of the enzyme-substrate and enzyme-product interaction". Binding pockets are similar and can be cross-validated, improving the reliability of reported binding affinities (**Figure 8C**). All in all, 2'-methylacetophenone and XDH form a stable complex.

## 4 Discussion

HUA is defined as a fasting blood uric acid level of more than 420 μmol/L on two different days in adults with a normal purine diet, irrespective of gender (8). It has a growing prevalence year by year and has become the second most prevalent metabolic disease in China, second only to diabetes mellitus (87). In addition to causing gout, elevated serum uric acid levels are associated with an increased risk of hypertension, cardiovascular disease, mortality, and progression of chronic kidney disease (88, 89). According to the mechanism of action, commercially available uric acid-lowering drugs mainly focus on inhibiting uric acid production and increasing uric acid excretion, which can also cause significant side effects on the human body in clinical practice (90, 91). Currently, safe, reliable and economical uric acid-lowering drugs are being developed (92). The high number of people with HUA worldwide is costly in terms of medical expenditure, and the dangers associated with poorly controlled development of gout are enormous (93). Consequently, HUA has become an important risk factor affecting the quality of life, and the prevention and control of HUA have become an important public health issue. It is worth every clinical worker to pay attention to it.

There is increasing recognition of the value of active ingredients in traditional Chinese medicine, and emerging evidence indicates that a number of herbal formulas can reduce uric acid production by inhibiting the activity of adenosine deaminase and (or) xanthine oxidase, or increase uric acid excretion by regulating the expression of uric acid transporter protein, and play a role in the pharmacological effect of lowering uric acid. Studies have shown that Paeonia veitchii Lynch (94) and turmeric (95) can inhibit xanthine oxidase activity and reduce blood uric acid levels. Dioscorea villosa (96, 97), Gardenia jasminoides (98) and Paeonia veitchii Lynch (94) can regulate the expression of urate transporter protein, reduce the reabsorption of uric acid, and promote the excretion of uric acid. ZP, a traditional Chinese herb and spice, has a powerful medicinal and dietary value. ZP has anti-inflammatory and analgesic effects, and studies have shown that peppercorns in ZP are known to activate the JAK2/STAT3 signaling pathway, which inhibits inflammation and reduces the production of pro-inflammatory cytokines such as IL-6 and TNFα, showing anti-inflammatory and analgesic a effects in a variety of animal models (13). A research study has found that extracts of peppercorn fruits have the effect of inhibiting xanthine oxidase activity *in vitro* (22), which may provide a new preventive and therapeutic direction in drug discovery for the prevention and treatment of HUA.

In this paper, with the support of computer-aided drug design and virtual drug screening, we have proposed a number of active ingredients and mechanisms of action that may be involved in ZP’s uric acid-lowering effects. This approach may enable more rapid development of new uric acid-lowering drugs and save human, material and financial resources; accelerate the speed of target discovery and improve the accuracy of target discovery to develop new drugs purposefully; and start from consideration of microscopic biomolecular structure, which can be more intuitive to understand and explain the experimental results showing ZP’s effects on uric acid-lowering.

With the TCMSP, UniProt and SwissTargetPrediction databases, we obtained the active ingredients of ZP and their corresponding targets and obtained a total of 101 active ingredients of ZP and 81 of its targets. Then we screened HUA-related targets through GeneCards, CTD and Open Target databases, and there were 140 HUA targets. Finally, we found 10 targets of hyperuricemic drugs through DrugBank.

The intersecting targets of ZP, hyperuricemia and drug were obtained by using Venny 2.1.0 and uploaded to the String database for PPI network mapping. The core targets included 15 genes including XDH, PTGS2, TNF, ESR1 and MAOA. In addition, we filtered and created a PPI map of “ZP-HUA-drug” using CytoNCA plug-in for Cytoscape v3.8.2. We examined degree values to explore the biological importance of proteins in a network, identifying nodes that are critical for communication within the network. Proteins with high values are important intermediate proteins that play important functional and kinetic roles, as well as serving as potential drug targets (99). From the Target sorting diagram, we found that XDH had the highest degree value among the core proteins. In mammals, catabolism of uric acid cannot function without Xanthine dehydrogenase. Xanthine dehydrogenase, also known as XDH or XO or XAN1, is an aerobic dehydrogenase enzyme with FAD (Flavin Adenine Dinucleotide) and FMN (Flavin Mononucleotide) as cofactors (100). This enzyme is involved in the metabolism of xanthine, converting it to uric acid by catalysis (101). It has been demonstrated that inhibition of XDH activity reduced blood uric acid levels (102). All of these are involved in the development of HUA, and XDH is an important protein in this PPI network that exerts a biological role in uric acid lowering as well as a potential drug target.

With the help of the DAVID database, we performed GO and KEGG pathway enrichment analyses on the intersected targets. The CC from the GO enrichment analysis graph reflected the localization of genes or proteins within the cell, with the plasma membrane and extracellular exosome enriched with the highest number of genes. It is well known that about 70% of uric acid in the body is excreted mainly through the kidneys (103, 104), and a large number of uric acid transporters are present in the membrane of renal cells, which reduce the level of uric acid in the blood through reabsorption. Some renal transporter proteins enhance the membrane transport of uric acid by facilitating ion transport as a driving force (10). In addition, the basic molecular alterations in the multi-omics networks of patients with gouty knee osteoarthritis involve acute inflammatory response, exosomes, immune response, lysosomes, linoleic acid metabolism and synthesis (105). Also, circulating exosomal miRNAs may be involved in the pathogenesis of nonalcoholic fatty liver disease and are associated with aminotransferase and uric acid (106). This suggests that the active ingredients of ZP may exert uric acid-lowering effects and functions in the plasma membrane and extracellular exosome.

KEGG pathway enrichment mainly includes metabolic pathways and lipid and atherosclerosis signaling pathways. We found the presence of uric acid-lowering active ingredient in ZP and the results showed that the active ingredient may be related to metabolic pathways and inflammatory pathways. The uric acid metabolic process is also involved in a variety of diseases of chronic inflammatory response. High uric acid in the blood will trigger the production of inflammatory bodies (107, 108). ZP’s active ingredients have analgesic and anti-inflammatory effect, and can reduce the inflammatory response brought about by high uric acid, and thus achieve the effect of controlling uric acid. IL-17 signaling pathway plays a key role in inflammation and autoimmune diseases (109). Studies have shown that uric acid injection in rats significantly increased renal injury, including renal tubule injury score, blood urea nitrogen, and serum creatinine, and at the same time induced the mass production of IL-17 and the recruitment of Th17 cells. Treatment of rats with a specific anti-IL-17 mAb mitigates urico-induced kidney injury with the inactivation of nuclear factor-κB. In conclusion, uric acid can induce the expression of IL-17 and activate the IL-17 signaling pathway, thereby leading to kidney injury. Neutralizing IL-17 can inhibit NF-κB signaling, thereby alleviating urico-induced kidney injury (110). The discovery that IL-17 signaling pathway is involved in hyperuricemia associated kidney injury provides a new perspective for understanding the role of uric acid in kidney injury. Advanced glycosylation end-product-receptor signaling pathway refers to the binding of protein glycosylation products (AGEs) to their receptor (RAGE) caused by hyperglycemia, Signaling pathways that trigger a series of reactions (111). High concentration of uric acid can increase the expression and exosecretion of high mobility group protein B1 (HMGB1) in endothelial cells, and HMGB1 interacts with advanced glycosylation end-product receptor (RAGE). Induced oxidative stress and inflammation (112) These mechanisms reveal the important role of uric acid in the occurrence of metabolic diseases and cardiovascular diseases, and provide a potential target for the treatment of multi-tissue organ damage caused by hyperuricemia.

Subsequently, we sought to clarify the mechanisms of the specific components of ZP, to find stable compound structures in ZP with drug-forming potential. We performed molecular docking of the potential active ingredients to the core target using Autodock Vina. The optimal binding sites of the ZP compounds to the urinary drug-lowering proteins were predicted using the Protein Plus platform. MD simulation was used to verify the stability of the optimal binding site. According to the molecular docking results, the core active ingredient of ZP was able to bind well to XDH, the target of the drug for HUA. A total of 49 optimal binding sites were identified for the peppercorn compounds and the urinary-lowering drug proteins using the Protein Plus platform, and four sites with the highest potential were selected based on druggability measures. Short 50 ns molecular simulations were performed for ligand-XDH complexes where the ligand was bound to the active site, and the HJ010-XDH complex was selected based on the high number of hydrogen bonding, and favorable RMSF, RMSD, Rg, and SASA properties. Finally, three longer MD simulations of 200 ns were carried out to further verify the binding stability of the site.

The results of the time-dependent RMSD analysis showed that all four systems (3xHJ ligand-protein complex simulations and the *apo* XDH) achieved equilibrium, although the ligands caused a greater shift to the overall structure compared to *apo* XDH. In contrast to existing studies on XDH, the values and magnitudes of RMSD values for simulated HJ-XDH complexes (between 0.184 and 0.260 nm) were consistent, and occasionally outperformed a variety of natural compounds: geraniol (0.39 nm) and lignans (0.27 nm) (113), and a potent XDH inhibitor: allopurinol (0.43 nm) (114). These differences may be a result of the simulation time, as longer simulations capture larger changes in protein structure.

Although the RMSD values were relatively stable, the structural compactness of the protein, as measured by the mean Rg and SASA, differed from another study, which showed that in XO, the Rg value was stable at around 2.88 nm and the SASA value was stable at about 326 nm^2^ and this result was similar to that of the XO- allopurinol complex (114). This differed considerably from our MD simulation results, probably because xanthine oxidase has two interconverted forms: xanthine dehydrogenase (XDH) and xanthine oxidase (XO), and therefore small differences were observed in the protein structure, thus leading our results to show higher Rg and SASA values as we simulated the XDH form. In the 200 ns simulation of HJ010 with XDH, the average Rg and SASA values of HJ010-XDH were almost all smaller than those of *apo* XDH, suggesting that the folding pattern of the protein did not undergo significant changes due to binding to the ligand, and therefore may remain stable throughout the simulation period.

RMSF values were also comparable to existing studies on MD simulations of similar enzymes. The *apo*-protein showed greater RMSF fluctuation compared to ligand-bound structures around peaks at Lys185 and Glu530 in our study, consistently echoing the findings from the ligand-bound and ligand-free studies on XO by Pan 2021 (114). As can be seen from the hydrogen bond analysis, HJ010, followed by HJ069, formed the largest number of polar contacts with XDH residues. Although the polar interactions within 50 ns simulations were volatile, compared with other ligands such as HJ028 and HJ059, a largely consistent pattern shows that the pockets have strong ligand interaction. The higher hydrophilicity of HJ010 may have contributed to the increased polar contacts. Further mutagenesis studies are needed to determine the role of these polar contacts on XDH inhibition. Three 200 ns MDS showed that the HJ010-XDH complex yielded favorable RMSD, RMSF and Rg values, while no separation of receptor and ligand was observed during all simulations. These results strongly suggested that the binding of the active compounds to their targets is stable and therefore these core compounds warrant further experimental investigation.

We have some limitations with regard to this article. In molecular docking, only rigid docking was selected, ignoring the flexibility of the receptor, and induced coordination docking or assembly docking can better understand the interaction between ligand and protein. In pharmacokinetics, although hepatotoxicity and carcinogenicity were assessed, other potential side effects, such as cardiovascular or renal toxicity, were not explored. Given that ZP may play a role in hyperuricemia induced multisystem damage, cardioToxCSM will be introduced later to predict cardioToxCSM (115) and renal toxicity of Extra - tree model (116). The research mainly relies on computational methods (docking, simulation and network pharmacology), and our next research goal will be to verify the molecular mechanism of ZP treatment of hyperuricemia from *in vivo* and *in vitro* models.

Based on bioinformatics analysis of network pharmacology, molecular docking and MD simulations, we propose that chemical constituents in ZP have a role in controlling uric acid and that these compounds could be viable candidates for future development of XDH inhibitors. The chemical composition of ZP is mainly divided into alkaloids, terpenoids, flavonoids, fatty acids and other chemical compounds (14), of which volatile oil has been a hot spot of research in recent years (117). The main chemical components of volatile oil and its content are affected by factors such as production area, harvesting time and extraction and processing techniques. At present, the development of ZP products in the market has great prospects, but first of all, it is necessary to solve the problem of the instability of the volatile oil of ZP. Only by maintaining the stability of the volatile oil of ZP can the loss of active chemicals be reduced so that they are not easily volatilized in food or medicine. In addition, understanding the pharmacological mechanism of action of the active substances of ZP *in vivo* can improve the success rate of product development and reduce the risks in clinical development. Our next task is to comprehensively validate the pharmacological mechanism of action of the active ingredients of ZP *in vivo* through experiments and to assess the biosafety of these ingredients, for which more clinical trials and comprehensive pharmacological and biochemical studies are necessary.

## 5 Conclusions

In this paper, a computational molecular modelling method is used to predict and calculate the interactions between potential active compounds of ZP with XDH, a target of HUA, which may become a uric acid-lowering drug. This will help clinical drug development and reduce time, effort and financial resources required to develop an effective medicinal treatment, giving new hope to a large number of patients with high uric acid and gout. Importantly, the compound we identified comes from a seasoning product with both medicinal and culinary value, a necessity in everyday diet that is safe, reliable and deeply culturally ingrained. We will continue to research and develop an understanding of the mechanism of controlling uric acid by ZP in depth, with subsequent research to be performed on cells and animals, so as to accumulate reference data for the development of derivatives of ZP, and to increase public awareness of its potential in the management of HUA. The capability to control uric acid through flexible intake of ZP in daily diet would be of immense benefit.

## Data Availability

The original contributions presented in the study are included in the article/**Supplementary Material**. Further inquiries can be directed to the corresponding authors.
