# Supplementary material for: Potential candidates from a functional food Zanthoxyli Pericarpium (Sichuan pepper) for the management of hyperuricemia: high-through virtual screening, network pharmacology and dynamics simulations
Source: Front Endocrinol (Lausanne). 2024 Dec 11;15:1436360. doi: 10.3389/fendo.2024.1436360 (PMC11668583; doi:10.3389/fendo.2024.1436360)
Supplement: Supplementary file 1 [file Table1.docx]

**Table S1** Details of 101 *Zanthoxyli Pericarpium* compounds obtained from the traditional Chinese medicine systems pharmacology database and analysis platform (TCMSP).

| **Compound code** | **Molecule Name** | **Mol ID** | **PubChem CID/SID** | **PubChem name** | **Canonical SMILES** |
| --- | --- | --- | --- | --- | --- |
| HJ001 | cis-linalol pyranoxide | MOL010265 | 6428574 | (3R,6R)-6-ethenyl-2,2,6-trimethyloxan-3-ol | CC1(C(CCC(O1)(C)C=C)O)C |
| HJ002 | cis-beta-Ocimene | MOL001110 | 5320250 | (Z)-beta-Ocimene | CC(=CCC=C(C)C=C)C |
| HJ003 | ZINC02140511 | MOL011544 | 1796220 | (+)-Longifolene | CC1(CCCC2(C3C1C(C2=C)CC3)C)C |
| HJ004 | (1S,2S)-2-isopropenyl-4-isopropylidene-1-methyl-1-vinylcyclohexane | MOL001168 | 6432312 | gamma-Elemene | CC(=C1CCC(C(C1)C(=C)C)(C)C=C)C |
| HJ005 | Cymol | MOL000117 | 7463 | P-Cymene | CC1=CC=C(C=C1)C(C)C |
| HJ006 | (L)-alpha-Terpineol | MOL000118 | 443162 | (-)-alpha-Terpineol | CC1=CCC(CC1)C(C)(C)O |
| HJ007 | ZINC02040970 | MOL000119 | 11241545 | (3R,6E)-nerolidol | CC(=CCCC(=CCCC(C)(C=C)O)C)C |
| HJ008 | 1,8-cineole | MOL000122 | 2758 | Eucalyptol | CC1(C2CCC(O1)(CC2)C)C |
| HJ009 | geraniol | MOL000123 | 637566 | Geraniol | CC(=CCCC(=CCO)C)C |
| HJ010 | o-Acetyltoluene | MOL001237 | 11340 | 2'-Methylacetophenone | CC1=CC=CC=C1C(=O)C |
| HJ011 | (-)-alpha-Pinene | MOL000125 | 440968 | (-)-alpha-Pinene | CC1=CCC2CC1C2(C)C |
| HJ012 | 4-isopropylcyclohex-2-en-1-one | MOL001252 | 642520 | (R)-4-Isopropylcyclohex-2-enone | CC(C)C1CCC(=O)C=C1 |
| HJ013 | (-)-nopinene | MOL000126 | 440967 | (-)-beta-Pinene | CC1(C2CCC(=C)C1C2)C |
| HJ014 | cis-nerolidol | MOL012609 | 12227246 | 1,6,10-Dodecatrien-3-ol, 3,7,11-trimethyl-, (3R,6Z)- | CC(=CCCC(=CCCC(C)(C=C)O)C)C |
| HJ015 | (E)-linalool oxide acetate pyr | MOL012618 | 6427501 | Linalool oxide acetate (pyranoid) | CC(=O)OC1CCC(OC1(C)C)(C)C=C |
| HJ016 | NERYLACETATE | MOL000128 | 1549026 | Geranyl acetate | CC(=CCCC(=CCOC(=O)C)C)C |
| HJ017 | linalyl anthranilate | MOL012866 | 23535 | Linalyl anthranilate | CC(=CCCC(C)(C=C)OC(=O)C1=CC=CC=C1N)C |
| HJ018 | (2S)-3-methoxypropane-1,2-diol | MOL013266 | 156179 | 1,2-Propanediol, 3-methoxy-, (S)- | COCC(CO)O |
| HJ019 | (E)-4-[(1R)-2,2-dimethyl-6-methylenecyclohexyl]but-3-en-2-one | MOL013267 | 11389922 | (-)-gamma-Ionone | CC(=O)C=CC1C(=C)CCCC1(C)C |
| HJ020 | cis-Pinene hydrate | MOL013268 | 1268143 | trans-2-Pinanol | CC1(C2CCC(C1C2)(C)O)C |
| HJ021 | CLOVENE | MOL013269 | 10102 | CID 10102 | CC1(C=CC23C1CCC(C2)(CCC3)C)C |
| HJ022 | Haplopine | MOL013270 | 165368 | CID 165368 | COC1=C2C=CC(=O)C(=C2NC3=C1C=CO3)OC |
| HJ023 | Kokusaginin | MOL013271 | 10227 | Kokusaginine | COC1=C(C=C2C(=C1)C(=C3C=COC3=N2)OC)OC |
| HJ024 | nerohdyl acetate | MOL013272 | 25021983 | N/A | CC(=CCCC(=CCCC(C)(C=C)OC(=O)C)C)C |
| HJ025 | Dymel A | MOL013273 | 8254 | Dimethyl Ether | COC |
| HJ026 | sanshool | MOL013274 | 6440935 | alpha-Sanshool | CC=CC=CC=CCCC=CC(=O)NCC(C)C |
| HJ027 | Schinifoline | MOL013275 | 133504 | Schinifoline | CCCCCCCC1=CC(=O)C2=CC=CC=C2N1C |
| HJ028 | (+)-Ledol | MOL001388 | 92812 | Ledol | CC1CCC2C1C3C(C3(C)C)CCC2(C)O |
| HJ029 | zoomaric acid | MOL001739 | 445638 | Palmitoleic acid | CCCCCCC=CCCCCCCCC(=O)O |
| HJ030 | Majudin | MOL001945 | 2355 | Bergapten | COC1=C2C=CC(=O)OC2=CC3=C1C=CO3 |
| HJ031 | L-Bornyl acetate | MOL000196 | 93009 | (-)-Bornyl acetate | CC(=O)OC1CC2CCC1(C2(C)C)C |
| HJ032 | Myrcene | MOL000197 | 31253 | Myrcene | CC(=CCCC(=C)C=C)C |
| HJ033 | (R)-linalool | MOL000198 | 443158 | (-)-Linalool | CC(=CCCC(C)(C=C)O)C |
| HJ034 | p-Ocimene | MOL000201 | 5281553 | beta-Ocimene | CC(=CCC=C(C)C=C)C |
| HJ035 | Moslene | MOL000202 | 7461 | gamma-Terpinene | CC1=CCC(=CC1)C(C)C |
| HJ036 | (6R)-6-isopropyl-3-methyl-1-cyclohex-2-enone | MOL000205 | 107561 | l-Piperitone | CC1=CC(=O)C(CC1)C(C)C |
| HJ037 | Methyleugenol | MOL000207 | 7127 | Methyleugenol | COC1=C(C=C(C=C1)CC=C)OC |
| HJ038 | OCT | MOL002137 | 356 | Octane | CCCCCCCC |
| HJ039 | BOX | MOL000219 | 20144841 | Hydron;benzoate | [H+].C1=CC=C(C=C1)C(=O)[O-] |
| HJ040 | beta-Gurjunene | MOL002335 | 6450812 | Beta-Gurjunene | CC1CCC2C(C2(C)C)C3C1CCC3=C |
| HJ041 | L-Limonen | MOL000234 | 439250 | (-)-Limonene | CC1=CCC(CC1)C(=C)C |
| HJ042 | Terragon | MOL002361 | 8815 | Estragole | COC1=CC=C(C=C1)CC=C |
| HJ043 | cis-Anethol | MOL002380 | 1549040 | cis-Anethole | CC=CC1=CC=C(C=C1)OC |
| HJ044 | alpha-humulene | MOL000024 | 5281520 | Humulene | CC1=CCC(C=CCC(=CCC1)C)(C)C |
| HJ045 | (-)-Comphene | MOL002453 | 440966 | (-)-Camphene | CC1(C2CCC(C2)C1=C)C |
| HJ046 | [(3S)-3,7-dimethyloct-6-enyl] acetate | MOL002455 | 6999975 | 6-Octen-1-ol, 3,7-dimethyl-, acetate, (3S)- | CC(CCC=C(C)C)CCOC(=O)C |
| HJ047 | eugenol | MOL000254 | 3314 | Eugenol | COC1=C(C=CC(=C1)CC=C)O |
| HJ048 | Skimmetin | MOL002558 | 5281426 | Umbelliferone | C1=CC(=CC2=C1C=CC(=O)O2)O |
| HJ049 | (-)-beta-Phellandrene | MOL000257 | 443161 | (-)-beta-Phellandrene | CC(C)C1CCC(=C)C=C1 |
| HJ050 | beta-Cubebene | MOL000266 | 93081 | beta-Cubebene | CC1CCC(C2C13C2C(=C)CC3)C(C)C |
| HJ051 | Skimmianin | MOL002663 | 6760 | Skimmianine | COC1=C(C2=C(C=C1)C(=C3C=COC3=N2)OC)OC |
| HJ052 | (1S,5S)-1-isopropyl-4-methylenebicyclo[3.1.0]hexane | MOL000268 | 11051711 | (-)-Sabinene | CC(C)C12CCC(=C)C1C2 |
| HJ053 | Diosmetin | MOL002881 | 5281612 | Diosmetin | COC1=C(C=C(C=C1)C2=CC(=O)C3=C(C=C(C=C3O2)O)O)O |
| HJ054 | beta-Selinene | MOL000035 | 442393 | beta-Selinene | CC(=C)C1CCC2(CCCC(=C)C2C1)C |
| HJ055 | CADINENE | MOL003534 | 10657 | beta-Cadinene | CC1=CCC2C(C1)C(CC=C2C)C(C)C |
| HJ056 | T-Muurolol | MOL003537 | 3084331 | T-Muurolol | CC1=CC2C(CCC(C2CC1)(C)O)C(C)C |
| HJ057 | spathulenol | MOL003571 | 13854256 | (+)-12-Epispathulenol | CC1(C2C1C3C(CCC3(C)O)C(=C)CC2)C |
| HJ058 | Anizol | MOL003579 | 7519 | Anisole | COC1=CC=CC=C1 |
| HJ059 | beta-sitosterol | MOL000358 | 222284 | Beta-Sitosterol | CCC(CCC(C)C1CCC2C1(CCC3C2CC=C4C3(CCC(C4)O)C)C)C(C)C |
| HJ060 | beta-caryophyllene | MOL000036 | 5281515 | Caryophyllene | CC1=CCCC(=C)C2CC(C2CC1)(C)C |
| HJ061 | Naphthalene, 1,2,3,4,4a,5,6,8a-octahydro-7-methyl-4-methylene-1-(1-methylethyl)-, (1alpha,4abeta,8aalpha)- | MOL003937 | 92313 | (-)-gamma-Cadinene | CC1=CC2C(CC1)C(=C)CCC2C(C)C |
| HJ062 | Cuminol | MOL003948 | 325 | 4-Isopropylbenzyl alcohol | CC(C)C1=CC=C(C=C1)CO |
| HJ063 | Perillen | MOL004294 | 68316 | Perillene | CC(=CCCC1=COC=C1)C |
| HJ064 | Hyperin | MOL004368 | 5281643 | Hyperoside | C1=CC(=C(C=C1C2=C(C(=O)C3=C(C=C(C=C3O2)O)O)OC4C(C(C(C(O4)CO)O)O)O)O)O |
| HJ065 | Otan | MOL004462 | 670 | Dihydroxyacetone | C(C(=O)CO)O |
| HJ066 | Azeton | MOL004472 | 180 | Acetone | CC(=O)C |
| HJ067 | Ayapanin | MOL004617 | 10748 | 7-Methoxycoumarin | COC1=CC2=C(C=C1)C=CC(=O)O2 |
| HJ068 | heptanoic acid | MOL004664 | 8094 | Heptanoic acid | CCCCCCC(=O)O |
| HJ069 | Ethyl geranate | MOL004665 | 5317247 | Ethyl geranate | CCOC(=O)C=C(C)CCC=C(C)C |
| HJ070 | anethole | MOL000475 | 637563 | Anethole | CC=CC1=CC=C(C=C1)OC |
| HJ071 | 1-alpha-Terpinyl acetate | MOL004788 | 93317 | 2-[(1S)-4-methylcyclohex-3-en-1-yl]propan-3-yl acetate | CC1=CCC(CC1)C(C)(C)OC(=O)C |
| HJ072 | HEX | MOL004918 | 8058 | n-HEXANE | CCCCCC |
| HJ073 | 3-methylhexane | MOL004971 | 13800357 | (-)-3-Methylhexane | CCCC(C)CC |
| HJ074 | Diosmin | MOL005093 | 5281613 | Diosmin | CC1C(C(C(C(O1)OCC2C(C(C(C(O2)OC3=CC(=C4C(=C3)OC(=CC4=O)C5=CC(=C(C=C5)OC)O)O)O)O)O)O)O)O |
| HJ075 | 3,4,5-trihydroxybenzoic acid | MOL000513 | 370 | Gallic Acid | C1=C(C=C(C(=C1O)O)O)C(=O)O |
| HJ076 | Nonacosane | MOL000514 | 12409 | Nonacosane | CCCCCCCCCCCCCCCCCCCCCCCCCCCCC |
| HJ077 | suberosin | MOL005795 | 68486 | Suberosin | CC(=CCC1=C(C=C2C(=C1)C=CC(=O)O2)OC)C |
| HJ078 | ()-Terpinen-4-ol | MOL000608 | 2724161 | (+)-Terpinen-4-ol | CC1=CCC(CC1)(C(C)C)O |
| HJ079 | delta-amorphene | MOL000615 | 441005 | (+)-delta-Cadinene | CC1=CC2C(CCC(=C2CC1)C)C(C)C |
| HJ080 | oleic acid | MOL000675 | 445639 | Oleic Acid | CCCCCCCCC=CCCCCCCCC(=O)O |
| HJ081 | palmitic acid | MOL000069 | 985 | Palmitic Acid | CCCCCCCCCCCCCCCC(=O)O |
| HJ082 | (R)-(-)-alpha-Phellandrene | MOL000698 | 442482 | (R)-(-)-alpha-Phellandrene | CC1=CCC(C=C1)C(C)C |
| HJ083 | WLN: VH6 | MOL000705 | 8130 | Heptanal | CCCCCCC=O |
| HJ084 | o-Cymol | MOL000712 | 10703 | O-Cymene | CC1=CC=CC=C1C(C)C |
| HJ085 | (-)-Citronellal | MOL000774 | 443157 | (S)-(-)-Citronellal | CC(CCC=C(C)C)CC=O |
| HJ086 | ascaridole | MOL008130 | 12308625 | p-Menth-2-ene, 1,4-epidioxy- | CC(C)C12CCC(C=C1)(OO2)C |
| HJ087 | T-BUTYLBENZENE | MOL008385 | 7366 | tert-Butylbenzene | CC(C)(C)C1=CC=CC=C1 |
| HJ088 | Butenone | MOL008722 | 6570 | Methyl vinyl ketone | CC(=O)C=C |
| HJ089 | Dodekan | MOL000885 | 8182 | Dodecane | CCCCCCCCCCCC |
| HJ090 | cineole | MOL000904 | N/A | N/A | N/A |
| HJ091 | beta-elemene | MOL000908 | 6918391 | beta-Elemene | CC(=C)C1CCC(C(C1)C(=C)C)(C)C=C |
| HJ092 | Terpilene | MOL000911 | 7462 | alpha-Terpinene | CC1=CC=C(CC1)C(C)C |
| HJ093 | tricyclene | MOL000913 | 55250308 | (1r)-(+)-Tricyclene | CC1(C2CC3C1(C3C2)C)C |
| HJ094 | Car-3-ene | MOL000916 | 442461 | (-)-3-Carene | CC1=CCC2C(C1)C2(C)C |
| HJ095 | cineole | MOL000917 | N/A | N/A | N/A |
| HJ096 | Mnk | MOL000924 | 8163 | 2-Undecanone | CCCCCCCCCC(=O)C |
| HJ097 | alpha-Farnesene | MOL000932 | 5281516 | alpha-Farnesene | CC(=CCCC(=CCC=C(C)C=C)C)C |
| HJ098 | 6,8-Nonadien-2-one, 8-methyl-5-(1-methylethyl)-, (S-(E))- | MOL009341 | 6451337 | Solanone | CC(C)C(CCC(=O)C)C=CC(=C)C |
| HJ099 | .alpha.-Carene | MOL009451 | 6430755 | (-)-2-Carene | CC1=CC2C(C2(C)C)CC1 |
| HJ100 | cuminal | MOL000974 | 326 | 4-Isopropylbenzaldehyde | CC(C)C1=CC=C(C=C1)C=O |
| HJ101 | quercetin | MOL000098 | 5280343 | Quercetin | C1=CC(=C(C=C1C2=C(C(=O)C3=C(C=C(C=C3O2)O)O)O)O)O |

N/A, not applicable.
